# Large-Scale Solvent-Free Chlorination of Hydroxy-Pyrimidines, -Pyridines, -Pyrazines and -Amides Using Equimolar POCl_3_

**DOI:** 10.3390/molecules17044533

**Published:** 2012-04-16

**Authors:** Han Wang, Kun Wen, Le Wang, Ye Xiang, Xiaocheng Xu, Yongjia Shen, Zhihua Sun

**Affiliations:** 1 School of Chemistry and Molecular Engineering, East China University of Science and Technology, Shanghai, 200237, China; 2 College of Chemistry and Chemical Engineering, Shanghai University of Engineering Science, Shanghai, 201620, China

**Keywords:** solvent-free, chlorination, phosphorous oxychloride (POCl_3_)

## Abstract

Chlorination with equimolar POCl_3_ can be efficiently achieved not only for hydroxypyrimidines, but also for many other substrates such as 2-hydroxy-pyridines, -quinoxalines, or even -amides. The procedure is solvent-free and involves heating in a sealed reactor at high temperatures using one equivalent of pyridine as base. It is suitable for large scale (multigram) batch preparations.

## 1. Introduction

The reaction of hydroxypyrimidines with phosphorous oxychloride (POCl_3_) is a simple procedure known for over 100 years and used widely in preparing chlorinated pyrimidine final products or intermediates for further transformations [1,2,3]. Over the years, the experimental conditions used for such chlorination reactions have changed little, and generally involve heating a hydroxy-containing substrate in excess POCl_3_ to reflux in the presence of an organic base. While such a protocol may be adequate for small scale synthesis in a research laboratory, it becomes an environmental burden to deal with the excess POCl_3_ in large scale preparations. Furthermore, even the process of quenching excess POCl_3_ in large scale needs safety attention due to the potential for latent exothermic events [4]. Therefore, improvements in reducing the amount of POCl_3_ used in large scale chlorination procedures would be welcomed for economic, environmental, and safety considerations [5,6].

We recently reported a protocol for large scale (milligram to kilogram batches) chlorination of hydroxypyrimidines using equimolar or less POCl_3_ with heating in a sealed reactor under solvent-free or low solvent conditions [7]. Our procedure has simple work up steps involving filtration or distillation, and generally gives high yields and purity of final products. Except one, the examples used in our original report are essentially all pyrimidine derivatives (single or fused ring systems). In this report, we sought to expand the scope of our original study by applying our procedure to a wider range of starting materials to probe the potential for generalization of this solvent-free protocol.

## 2. Results and Discussion

Because our previous report only covered four examples of single-ring hydroxypyrimidines, we tested six more pyrimidines here with mono-chloro, bromo, methyl, or amino substitution at various positions. All the reactions were carried out at a scale of 0.3 moles (~30–60 g of pyrimidine starting materials) with equimolar POCl_3_ per OH group and one equivalent of pyridine as base. The reactions were performed in sealed reactors for 2 hours as before, but at 160 °C instead of the 180 °C used in our original procedure. Despite the slightly lower reaction temperature, all trials gave satisfactory results (>80% isolated yields), as summarized in Table 1. 

**Table 1:** Chlorination of hydroxypyrimidines at 0.3 mole scale. 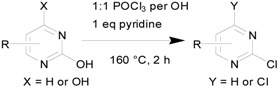

Entry	Starting Material	Scale	Product	Yield	Lit. Yield (Scale, Time)
**1**	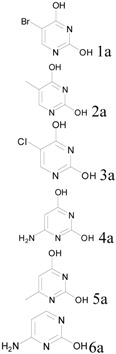	57 g	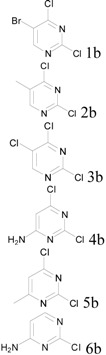	91%	88% (10 g, 1.7 hours) [8]
**2**		38 g		88%	92% (252 g, 20 hours) [9]
**3**		44 g		89%	69% (25 g, 3 hours) [10]
**4**		38 g		85%	27% (10 g, 10 hours) [11]
**5**		38 g		95%	70% (10 g, 3 hours) [12]
**6**		33 g		83%	59% (alternative reaction) [13]

In these trials, liquid final products were isolated by distillation after extraction (entries 1–3,5) and solid products (entries 4 and 6) were isolated by filtration after quenching the reaction with cold water. For comparison with literature reported results, we sought to compare our results with those with similar reaction scales and using POCl_3_ as reagent. As shown in Table 1, our protocol produced comparable results to others (entries 1 and 2) and sometimes better yields than reported cases.

**Figure 1:**
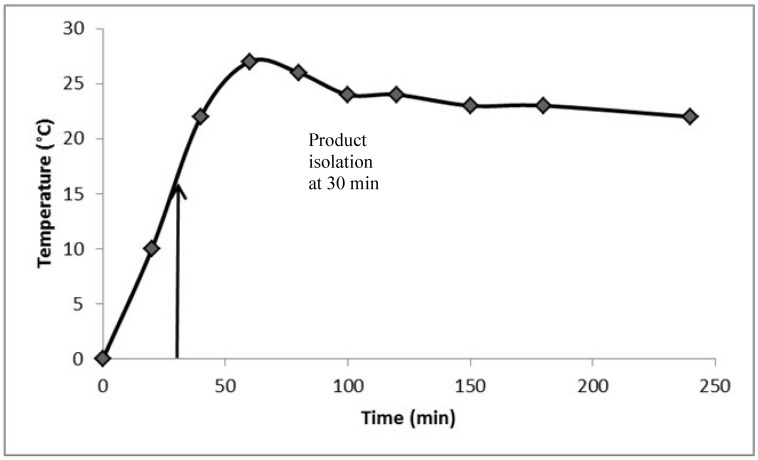
Temperature change after quenching a 100-gram scale reaction.

Given the safety concerns of quenching large scale POCl_3_ as studied in a recent report [4], we also investigated the event of quenching in our protocol. After the completion of reaction, due to the efficiency of our protocol and the fact that no excess of POCl_3_ was used, we expect that quenching of the reaction mixture will not involve hydrolysis of significant amount of POCl_3_ itself. Therefore, no significant exotherm was of concern upon initial quenching of the reaction. Instead, we believe that quenching of species such as phosphorodichloridic acid as a possible end product was the major event [4]. This was supported by the low and delayed heat release during the first hour of quenching of a large scale reaction with cold water (see Figure 1), by which time the desired product was already separated from the quenching solution. In the particular example shown in Figure 1, 1.4 mole of POCl_3_ was used in the reaction and the final reaction mixture was quenched with 500 mL of cold water. This quenching ratio was smaller than the quenching process detailed in a report by Amgen scientists [4], yet the temperature raise was also lower in our case. Therefore, any delayed heat release due to the hydrolysis of phosphorodichloridic acid and alike could be safely handled aside without significantly affecting the isolation of the desired products. 

Next, our attention was turned to chlorination of pyridine derivatives. We used seven 2-hydroxypyridines and one quinolone. It was interesting to discover that the reactions could be efficiently carried out at an even lower temperature of 140 °C. Furthermore, no additional pyridine was needed for reactions involving 2-hydroxypyridines, as all the pyridine starting materials can effectively act as base. For the reaction with quinolone, 0.6 equivalents of pyridine were added. The reaction scales and yields were summarized in Table 2.

**Table 2:** Chlorination of 2-hydroxypydridines at 0.5 mole scale. 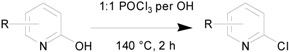

Entry	Starting Material	Scale	Product	Yield	Lit. Yield (Scale, Time)
**1**	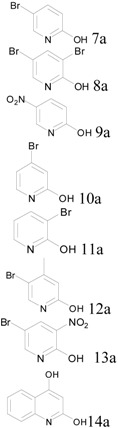	87 g	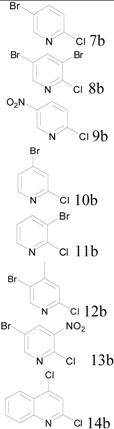	95%	49–83% * [14,15]
**2**		127 g		92%	66% * [16]
**3**		70 g		93%	66%, 89% (12 g, 3 hours) [17,18]
**4**		87 g		91%	52% * [19]
**5**		87 g		90%	65% * [20]
**6**		94 g		91%	48–50% * [21,22]
**7**		109 g		93%	65% (3.1 g, overnight) [23]
**8**		81 g		88%	94% (2 g, 24 hours) [24]

*: alternative reactions.

All reactions for pyridines were performed at 0.5 moles scale, with 70–126 g of hydroxyl pyridine or quinoline starting materials. All trials for the pyridine substrates gave 90% or more isolated yields. Except for entry 4 in Table 2 where the product was isolated by distillation after extraction with ethyl acetate, all other products were solids and were isolated using filtration after quenching the reaction with cold water and adjusting pH to 8–9. For pyridine analogous, few reported examples used POCl_3_ for chlorination. For those that did, our method is comparable or better (entries 3 and 7, Table 2). Compared to reported synthesis using alternative protocols, our method is generally better in terms of isolated yields. Entry 8 of Table 2 also showed an interesting case of good atom economy. There were two OH groups in the quinolone, and only 0.5 equivalent of POCl_3_ per OH was used. Yet, the isolated yield was very good at 88% for the dichloro product, indicating that each POCl_3_ provided more than one chlorine atom to form the final product. This result is consistent with findings in our original report that in some cases even substoichiometric amount of POCl_3_ was sufficient to produce respectable results using our solvent-free protocol [7]. 

The third class of starting materials we used were benzopyrazines (quinoxalines) or pyridopyrazine. Four different starting materials with either one or two hydroxyl groups were tested using our protocol at 0.3 moles scale. In those cases, high yield conversion required conditions similar to that of the pyrimidine cases, with one equivalent POCl_3_ per OH group, one equivalent of pyridine, and heating at 160 °C for 2 hours. The results were summarized in Table 3. All final products were isolated by filtration after quenching the reactions.

**Table 3:** Chlorination of hydroxyl benzopyrazions and pyridopyrazine (0.3 mole scale). 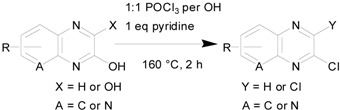

Entry	Starting Material	Scale	Product	Yield	Lit. Yield (Scale, Time)
**1**	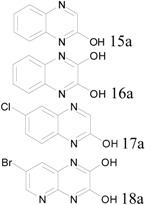	44 g	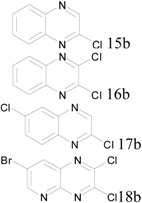	94%	56% (60 g, 2 hours) [25] 88% (15 g, 0.7 hour) [26]
**2**		49 g		96%	93% (64 g, 4 hours) [27]
**3**		54 g		86%	40% (164 g, 0.5 hour) [28]
**4**		73 g		94%	5% (6 g, 1.5 hours) [29]

**Scheme 1:**
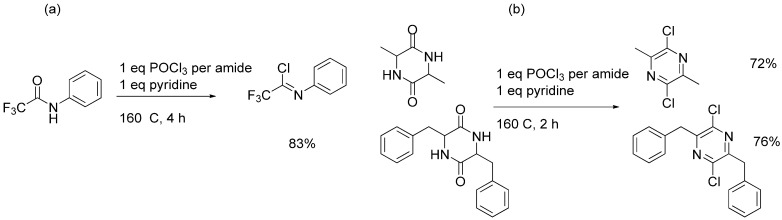
Chlorination of amides at 0.3 mole scale.

Given the excellent results of chlorination for the above three classes of aromatic starting materials, we thought about the possibility of using the same procedure of chlorination to convert a normal amide to an imidoylchloride, but imidoylchlorides are generally not stable enough for aqueous workup and purification. Therefore, we choose to test three examples where the imidoylchloride is either stable or can be further transformed during the reaction to another stable compound (Scheme 1). The first example was the conversion of *N*-trifluoroacetylaniline to the corresponding imidoylchloride, which can be isolated. The conversion achieved 83% isolated yield after 4 hours of reaction. However, unlike previous cases of product isolation through either distillation of filtration, the imidoylchloride needs to be purified by silica gel chromatography. In literature reporting synthesis of the same compound, an alternative method was used and a 66% yield was achieved [30]. Similarly, two 2,5-piperazinediones (dimeric condensation product from α-amino acids) were treated using the same protocol for 2 hours and were converted to the corresponding pyrazines instead of imidoylchlorides in 72–76% yields after purification by silica gel chromatography. Again, these results compare favourably with literature reported results of 25% and 41% for the two dichloropyrazines [31,32].

## 3. Experimental

### 3.1. General

All reactions were carried out in Teflon-lined stainless steel reactors. Analytical HPLC-MS (6130 Single Quandrupole LC/MS system, Aglilent, Santa Clara, CA, USA) was performed using a C18 reverse phase column (5 μm, 4.6 × 250 mm) with aqueous TFA and CH_3_CN gradients monitored at 230 nm for purity and detected with electrospray ionization (ESI) single quadruple mass spectrometer (6130 Single Quandrupole LC/MS system, Aglilent, Santa Clara, CA, USA) for MS. NMR was recorded at 400 MHz for ^1^H and 100 MHz for ^13^C. All known compounds were positively identified with MS and satisfactory changes in ^1^H or ^13^C-NMR (ARX400, Bruker, Fällanden, Switzerland) from starting materials. Melting points (mp) were determined with a WRS-1B digital melting-point apparatus (Shanghai, China).

### 3.2. Procedures for Chlorination of Pyrimidines, Benzo- and Pyrido-pyrazines, and Amides

To a 150 mL Teflon-lined stainless steel reactor was added the hydroxyl-containing substrate (0.3 moles), POCl_3_ (0.3 or 0.6 moles, 1 equivalent POCl_3_ per reactive OH or amide group), and pyridine (0.3 moles). After closing the reactor, the reaction mixture was heated to 160 °C for 2 hours (4 hours for trifluoroacetylaniline). After cooling, the reactor was carefully opened, and the contents were quenched with cold water (~0 °C, 100 mL), and the solution’s pH was adjusted to 8–9 with saturated Na_2_CO_3_ solution. For liquid sample, it was extracted with ethyl acetate, dried, removed solvent, and then distilled. Solid products were collected after filtration, and washing with a small amount of methyl *t*-butyl ether, and dried. Amide substrates that gave either imidoylchloride or pyrazine were extracted with 100 mL ethyl acetate, dried, and purified on a silica gel column after solvent removal. 

### 3.3. Pyrimidine Products

*2*,*4-Dichloro-5-bromopyrimidine* (**1b**). Obtained from 2,4-dihydroxy-5-bromopyrimidine (57.3 g), POCl3 (54 mL), and pyridine (24.3 mL) after distillation (110–112 °C/760 mm Hg). Yield: 61.9 g, 90.5%; Purity: 98%; ^1^H-NMR (CDCl_3_, δ, ppm): 8.69 (s, 1H). ^13^C-NMR (CDCl_3_, δ, ppm): 161.6; 161.4; 158.9; 118.8. MS (ESI, *m/z*): 226.9.

*2*,*4-Dichloro-5-methylpyrimidine* (**2b**). Obtained from 2,4-dihydroxy-5-methylpyrimidine (37.8 g), POCl_3_ (54 mL), and pyridine (24.3 mL) after distillation (114–116 °C/1 mm Hg). Yield: 43.0 g, 88%; Purity: 98%; ^1^H-NMR (CDCl_3_, δ, ppm):8.36 (s, 1H), 2.30(d, *J =* 0.4 Hz, 3H); ^13^C-NMR (CDCl_3_, δ, ppm):162.3, 160.0, 157.9, 129.0, 15.7; MS (ESI, *m/z*): 163.1.

*2*,*4*,*5-Trichloropyrimidine* (**3b**). Obtained from 2,4-dihydroxy-5-chloropyrimidine (44.1 g), POCl_3_ (54 mL) and pyridine (24.3 mL) after distillation (94–96 °C/12 mm Hg). Yield: 48.9 g, 89%; Purity: 96%; ^1^H-NMR (CDCl_3_, δ, ppm):8.59 (s, 1H); ^13^C-NMR (CDCl_3_, δ, ppm): 159.9, 158.7, 157.9, 129.2; MS (ESI, *m/z*): 183.0.

*2*,*4-Dichloro-6-aminopyrimidine* (**4b**). Obtained from 6-amino-2,4-dihydroxypyrimidine (38 g), POCl_3_ (54 mL) and pyridine (24.3 mL) after filtration. Yield: 41.8 g, 85%; Purity: 97%; m.p.: 253–254 °C; ^1^H-NMR (DMSO, δ, ppm): 7.75 (d, br, *J* *= *32 Hz, 2H), 6.45(s, 1H); ^13^C-NMR (DMSO, δ, ppm): 166.4, 159.4, 158.4, 102.2; MS (ESI, *m/z*): 164.1.

*2*,*4-Dichloro-6-methylpyrimidine* (**5b**). Obtained from 2,4-dihydroxy-6-methylpyrimidine (37.8 g), POCl_3_ (54 mL) and pyridine (24.3 mL) after distillation (98–100 °C/1 mm Hg). Yield: 41.8 g, 85%; Purity: 98%; m.p.: 46–47 °C; ^1^H-NMR (CDCl_3_, δ, ppm): 7.19 (d, *J = *2.8 Hz, 1H), 2.54(d, *J = *4.4 Hz, 3H); ^13^C-NMR (CDCl_3_, δ, ppm): 171.8, 162.3, 160.4, 119.4, 23.8; MS (ESI, *m/z*): 163.1.

*2-Chloro-6-aminopyrimidine* (**6b**). Obtained from 6-amino-2-hydroxypyrimidine (33 g), POCl_3_ (27 mL) and pyridine (24.3 mL) after filtration. Yield: 31.0 g, 83%; Purity: 97%; m.p.: 205–207 °C; ^1^H-NMR (CDCl_3_, δ, ppm): 7.94 (d, *J* *= *6 Hz, 1H), 7.39 (s, br, 2H), 6.39 (d, *J* *= *5.6 Hz, 1H); ^13^C-NMR (CDCl_3_, δ, ppm): 165.7, 160.3, 157.2, 104.5; MS (ESI, *m/z*): 130.1.

### 3.4. Quinoxaline and Pyridopyrazine Products

*2-Chloroquinoxaline* (**15b**). Obtained from 2-hydroxyquinoxaline (43.8 g), POCl_3_ (27 mL) and pyridine (24.3 mL) after filtration. Yield: 48.5 g, 94%; Purity: 98%; m.p.: 48–50 °C; ^1^H-NMR (CDCl_3_, δ, ppm): 8.78 (s, 1H), 8.07 (m, 1H), 7.97 (m, 1H), 7.75 (m, 2H); ^13^C-NMR (CDCl_3_, δ, ppm): 147.3, 144.9, 141.9, 140.9, 131.1, 130.1, 129.3, 128.5; MS (ESI, *m/z*): 165.1.

*2*,*3-Chloroquinoxaline* (**16b**). Obtained from 2,3-dihydroxyquinoxaline (48.6 g), POCl_3_ (54 mL) and pyridine (24.3 mL) after filtration. Yield: 57.3 g, 96%; Purity: 97%; m.p.: 148–150 °C; ^1^H-NMR (CDCl_3_, δ, ppm): 8.04 (m, 2H), 7.82 (m, 2H); ^13^C-NMR (CDCl_3_, δ, ppm): 145.4, 140.6, 131.2, 128.2; MS (ESI, *m/z*): 200.1.

*2*,*6-Chloroquinoxaline* (**17b**). Obtained from 2-hydroxy-6-chloroquinoxaline (54.2 g), POCl_3_ (27 mL) and pyridine (24.3 mL) after filtration. Yield: 51.4 g, 86%; Purity: 98%; m.p.: 156–158 °C; ^1^H-NMR (CDCl_3_, δ, ppm): 8.04 (m, 2H), 7.82 (m, 2H); ^13^C-NMR (CDCl_3_, δ, ppm): 145.4, 140.6, 131.2, 128.2; MS (ESI, *m/z*): 199.0.

*7-Bromo-2,3-dichloropyrido[2,3-b]pyrazine* (**18b**). Obtained from 2,3-dihydroxy-7-bromo pyrido[2,3-b]pyrazine (73.0 g), POCl_3_ (54 mL) and pyridine (24.3 mL) after filtration. Yield: 79.0 g, 94%; Purity: 98%; m.p.: 138–140 °C; ^1^H-NMR (CDCl_3_, δ, ppm): 9.17 (d, *J* *= *2.4 Hz, 1H), 8.57 (d, *J* *= *2.4 Hz, 1H); ^13^C-NMR (CDCl_3_, δ, ppm): 156.1, 149.0, 147.8, 147.2, 138.6, 136.2, 122.4; MS (ESI, *m/z*): 277.9.

### 3.5. Imidoylchloride and Pyrazine Products

*N-Phenyltrifluoroacetimidoyl chloride*. Obtained as an oil from trifluoroacetyl aniline (56.7 g), POCl_3_ (27 mL) and mL pyridine (24.3) after silica gel chromatography (eluent: petroleum ether/ethyl acetate 20:1). Yield: 51.7 g, 83%; Purity: 98%; ^1^H-NMR (CDCl_3_, δ, ppm): 7.48 (m, 2H), 7.34 (m, 1H), 7.13 (d, *J* *= *8 Hz, 2H); ^13^C-NMR (CDCl_3_, δ, ppm): 143.5, 131.9, 129.1 (2C), 127.4, 120.7 (2C), 117.5; ^19^F-NMR (CDCl_3_, δ, ppm): −71.6; MS (ESI, *m/z*): 208.1.

*2*,*5-Dichloro-3*,*6-dimethylpyrazine*. Obtained as a white solid from 3,6-dimethylpiperazine-2,5-dione (42.6 g), POCl_3_ (54 mL) and pyridine (24.3 mL) after silica gel chromatography (eluent: petroleum ether/ethyl acetate 10:1). Yield: 38.2 g, 72%; Purity: 98%; m.p.: 63–65 °C; ^1^H-NMR (CDCl_3_, δ, ppm): 2.60 (s, 6H); ^13^C-NMR (CDCl_3_, δ, ppm): 149.8 (2C), 145.6 (2C), 29.7, 21.3; MS (ESI, *m/z*): 177.1.

*2*,*5-Dichloro-3*,*6-dibenzylpyrazine.* Obtained as a white solid from 3,6-dibenzylpiperazine-2,5-dione (88.2 g), POCl_3_ (54 mL) and pyridine (24.3 mL) after silica gel chromatography (eluent: petroleum ether/ethyl acetate 15:1). Yield: 74.0 g, 76%; Purity: 98%; m.p.: 109–111 °C; ^1^H-NMR (CDCl_3_, δ, ppm): 7.29 (m, 10H), 4.27 (s, 4H); ^13^C-NMR (CDCl_3_, δ, ppm): 152.1, 145.8, 136.4, 129.1, 128.6, 127.0, 40.4; MS (ESI, *m/z*): 330.1.

### 3.6. Procedures for Chlorination of 2-Hydroxypyridines and 2,4-Dihydroxyquinoline

To a 150 mL Teflon-lined stainless steel reactor was added the hydroxy-containing substrate (0.5 moles) and POCl_3_ (0.5 moles; for quinoline starting material, 0.3 mole of pyridine was also used). After closing the reactor, the reaction mixture was heated to 140 °C for 2 hours. After cooling, the reactor was carefully opened, and the content was quenched with 100 mL cold water (~0 °C), and the solution’s pH was adjusted to 8–9 with saturated Na_2_CO_3_ solution. The solid product was precipitated out and collected after filtration, washed with a small amount of methyl *t*-butyl ether, and dried. For liquid sample, it was extracted with ethyl acetate, dried, removed solvent, and then distilled.

*2-Chloro-5-bromopyridine* (**7b**). Obtained from 2-hydroxy-5-bromopyridine (87.0 g) and POCl_3_ (45 mL) after filtration. Yield: 91.0 g, 95%; Purity: 97%; m.p.: 69–71 °C; ^1^H-NMR (CDCl_3_, δ, ppm): 8.48 (d, *J* *= *2.4 Hz, 1H), 7.79 (dd, *J* *= *2.4 Hz, *J* *= *8.4 Hz, 1H), 7.26 (d, *J* *=* 8.4 Hz, 1H); ^13^C-NMR (CDCl_3_, δ, ppm): 150.7, 150.1, 141.2, 125.6, 119.1; MS (ESI, *m/z*): 192.0.

*2-Chloro-3*,*5-dibromopyridine* (**8b**). Obtained from 2-hydroxy-3,5-dibromopyridine (126.5 g) and POCl_3_ (45 mL) after filtration. Yield: 124.7 g, 92%; Purity: 97%; m.p.: 41–43 °C; ^1^H-NMR (CDCl_3_, δ, ppm): 8.38 (d, *J = *2.4 Hz, 1H), 8.06 (t, *J* *= *2 Hz, 1H); ^13^C-NMR (CDCl_3_, δ, ppm): 149.6, 148.8, 144.0, 120.8, 118.7; MS (ESI, *m/z*): 269.9.

*2-Chloro-5-nitropyridine* (**9b**). Obtained from 2-hydroxy-5-nitropyridine (70.0 g) and POCl_3_ (45 mL) after filtration. Yield: 73.5 g, 93%; Purity: 98%; m.p.: 109–111 °C; ^1^H-NMR (CDCl_3_, δ, ppm): 9.25 (d, *J* *=* 2.8 Hz, 1H), 8.47 (dd, *J* *= *2.8 Hz, *J* *= *8.8 Hz, 1H), 7.57 (d, *J* *= *8.8 Hz, 1H); ^13^C-NMR (CDCl_3_, δ, ppm): 157.1, 145.4, 143.4, 133.6, 124.8; MS (ESI, *m/z*): 159.1.

*2-Chloro-4-bromopyridine* (**10b**). Obtained from 2-hydroxy-4-bromopyridine (87.0 g) and POCl_3_ (45 mL) after distillation (108–110 °C/0.5 mm Hg). Yield: 87.0 g, 91%; Purity: 98%; ^1^H-NMR (CDCl_3_, δ, ppm): 8.21 (d, *J* *= *5.6 Hz, 1H), 7.51 (d, *J* *= *5.6 Hz, 1H), 7.38 (dd, *J* *= *1.6 Hz, *J* *= *5.2 Hz, 1H); ^13^C-NMR (CDCl_3_, δ, ppm): 147.4, 145.3, 129.4, 122.6, 121.1; MS (ESI, *m/z*): 192.0.

*2-Chloro-3-bromopyridine* (**11b**). Obtained from 2-hydroxy-3-bromopyridine (87.0 g) and POCl_3_ (45 mL) after filtration. Yield: 86.0 g, 90%; Purity: 96%; m.p.: 54–56 °C; ^1^H-NMR (CDCl_3_, δ, ppm): 8.35 (td, *J* *= *1.2 Hz, *J* *=* 8 Hz, 1H), 7.95 (dd, *J* *=* 1.2 Hz, *J* *= *8 Hz, 1H), 7.14 (dd, *J* *= *8 Hz, *J* *=* 4.4 Hz, 1H); ^13^C-NMR (CDCl_3_, δ, ppm): 150.9, 147.9, 142.2, 123.3, 120.4; MS (ESI, *m/z*): 192.0.

*2-Chloro-4-methyl-5-bromopyridine *(**12b**). Obtained from 2-hydroxy-4-methyl-5-bromopyridine (94.0 g) and POCl_3_ (45 mL) after filtration. Yield: 94.0 g, 91%; Purity: 97%; m.p.: 115–117 °C; ^1^H-NMR (CDCl_3_, δ, ppm): 8.43 (dd, *J* *=* 14 Hz, *J* *=* 7.2 Hz, 1H), 7.23 (td, *J* *=* 6.4 Hz, 1H), 2.39 (dd, *J* *=* 6.4 Hz, *J* *= *12 Hz, 3H); ^13^C-NMR (CDCl_3_, δ, ppm): 150.8, 150.8, 150.2, 149.9, 126.0, 122.0; MS (ESI, *m/z*): 206.0.

*2-Chloro-3-nitro-5-bromopyridine* (**13b**). Obtained from 2-hydroxy-3-nitro-5-bromopyridine (109.0 g) and POCl_3_ (45 mL) after filtration. Yield: 110.0 g, 93%; Purity: 97.5%; m.p.: 67–68 °C; ^1^H-NMR (CDCl_3_, δ, ppm): 8.71 (d, *J* *=* 2 Hz, *J* *= *7.2 Hz, 1H), 8.38 (d, *J* *= *2 Hz, 1H); ^13^C-NMR (CDCl_3_, δ, ppm): 153.4, 144.6, 142.1, 136.5, 118.8; MS (ESI, *m/z*): 239.1.

*2*,*4-Dichloroquinoline* (**14b**). Obtained from 2,4-dihydroxyquinoline (81.0 g), POCl_3_ (45 mL) and pyridine (24.3 mL) after filtration. Yield: 79.0 g, 88%; Purity: 97%; m.p.: 188–190 °C; ^1^H-NMR (CDCl_3_, δ, ppm): 8.15 (d, *J* = 8.4 Hz, 1H), 8.02 (d, *J* *= *8.4 Hz, 1H), 7.78 (td, *J* *= *7.2 Hz, *J* *=* 1.2 Hz, 1H), 7.63 (td, *J* *=* 7.2 Hz, *J* *= *1.2 Hz, 1H), 7.47 (d, *J* *= *2 Hz, 1H); ^13^C-NMR (CDCl_3_, δ, ppm): 149.8, 148.1, 144.3, 131.5, 129.0, 127.8, 125.1, 124.1, 121.9; MS (ESI, *m/z*): 198.1.

## 4. Conclusions

A variety of hydroxylated nitrogen-containing heterocycles as well as amides can be efficiently chlorinated using equimolar POCl_3_ in a sealed reactor for just 2 hours at 140–160 °C using pyridine as a base. It seems that the protocol is broadly applicable to many substrates and works equally well or better in terms of yield, less reagent, and shorter reaction time than conventional protocols that involve heating to reflux in POCl_3_. It is quite suitable for large scale preparation of the relevant chlorinated products.

## Supplementary Materials

Supplementary materials can be accessed at: http://www.mdpi.com/1420-3049/17/4/4533/s1.
