# Supplementary material for: Large-Scale Solvent-Free Chlorination of Hydroxy-Pyrimidines, -Pyridines, -Pyrazines and -Amides Using Equimolar POCl3
Source: Molecules. 2012 Apr 16;17(4):4533–44. doi: 10.3390/molecules17044533 (PMC6290571; doi:10.3390/molecules17044533)
Supplement: Supplementary file 1 [file molecules-17-04533-s001.pdf]

Supporting Information

Compound 1b

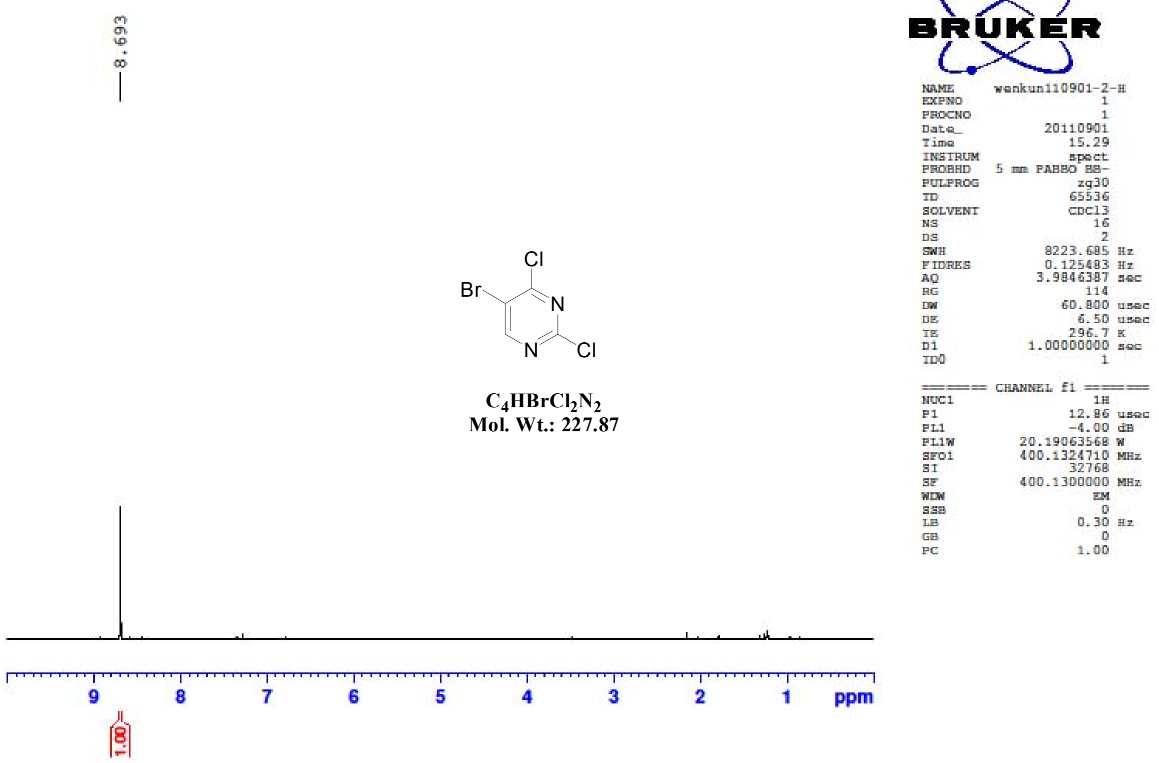

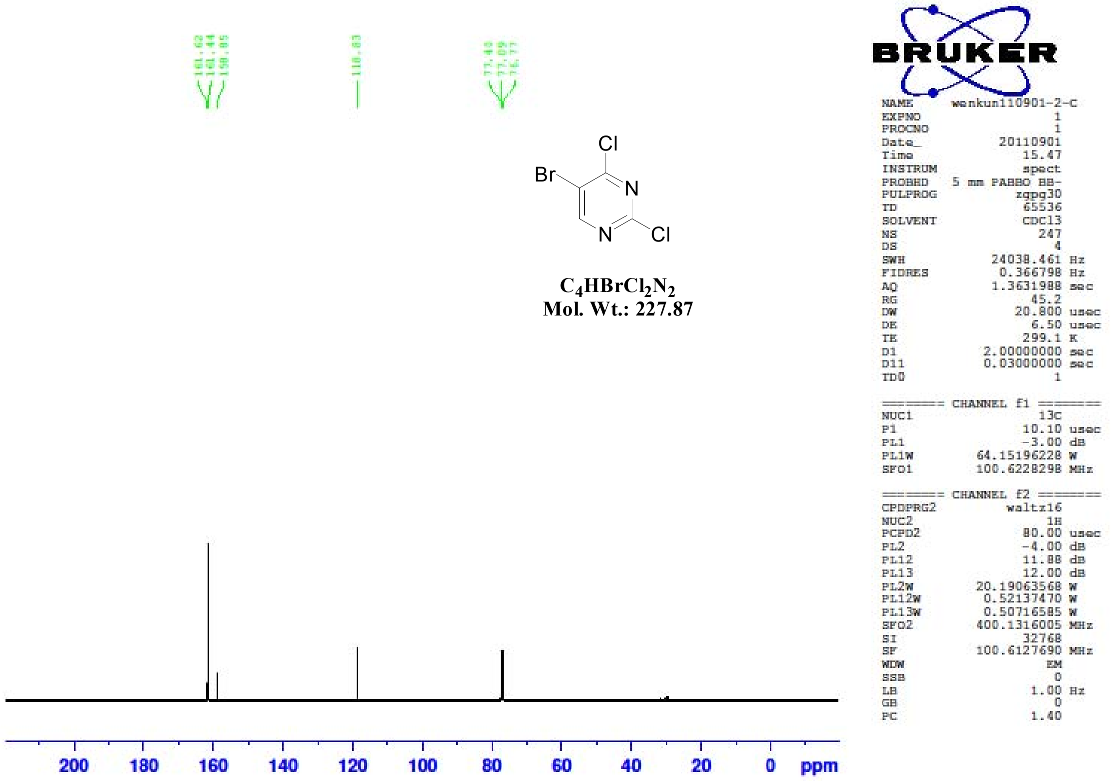

Compound 2b

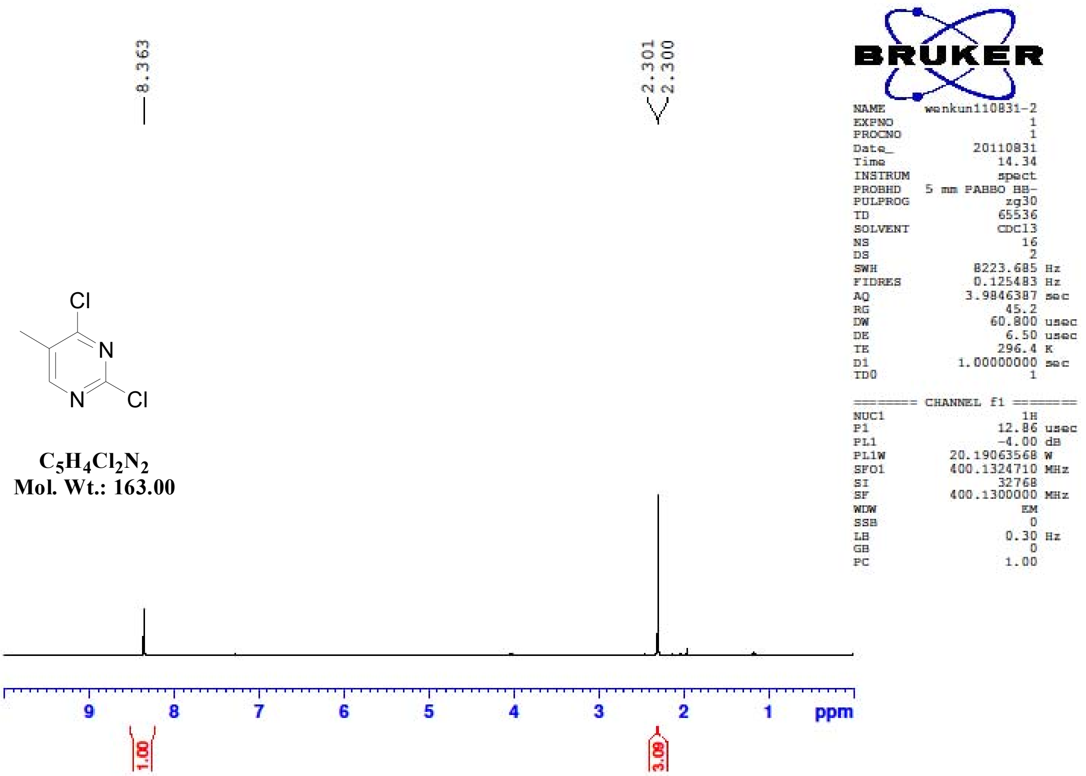
8.363
2.301
2.300
Cl
N
N
Cl
C5H4Cl2N2
Mol. Wt.: 163.00
BRUKER
NAME wenkun110831-2
EXPNO 1
PROCNO 1
Date_ 20110831
Time 14.34
INSTRUM spect
PROBHD 5 mm PABBO BB-
PULPROG zg30
TD 65536
SOLVENT CDCl3
NS 16
DS 2
SWH 8223.685 Hz
FIDRES 0.125483 Hz
AQ 3.9846387 sec
RG 45.2
DW 60.800 usec
DE 6.50 usec
TE 296.4 K
D1 1.00000000 sec
TD0 1
======= CHANNEL f1 =======
NUC1 1H
P1 12.86 usec
PL1 -4.00 dB
PL1W 20.19063568 W
SFO1 400.1324710 MHz
SI 32768
SF 400.1300000 MHz
WDW EM
SSB 0
LB 0.30 Hz
GB 0
PC 1.00
9
8
7
6
5
4
3
2
1
ppm
1.00
3.09

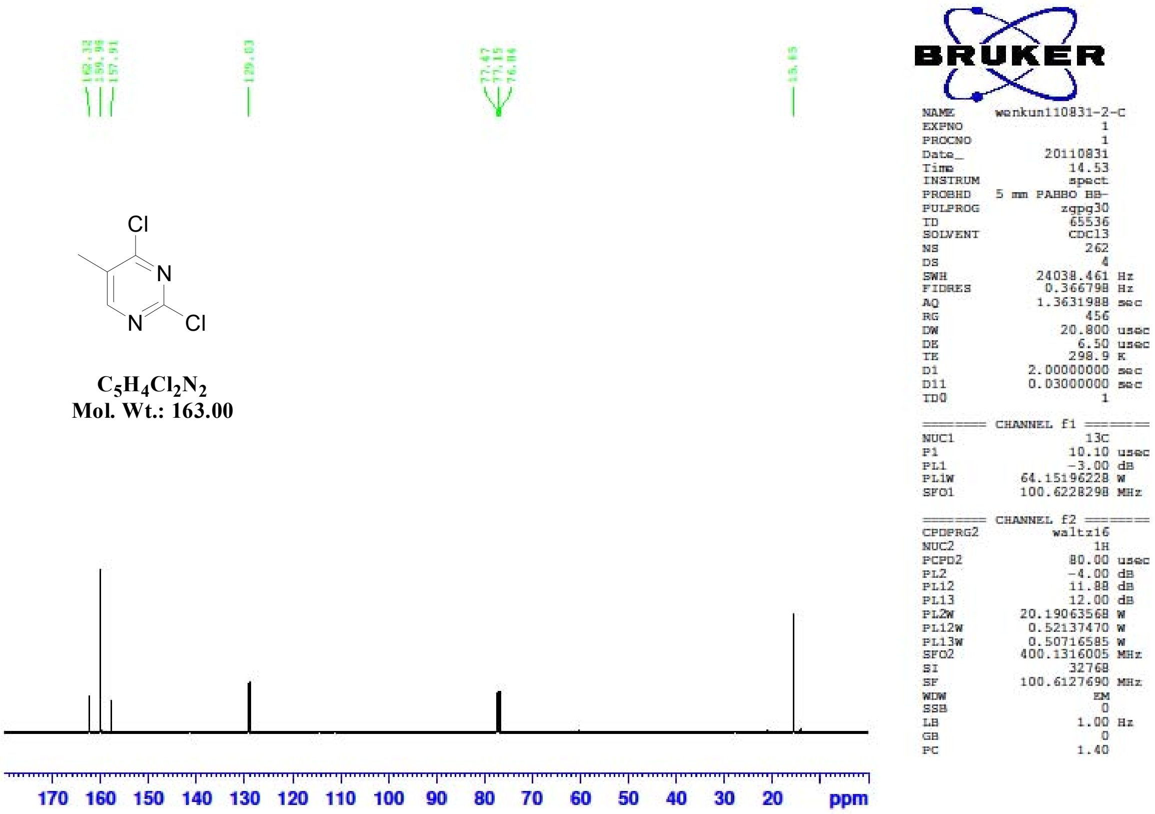
162.32
159.98
157.91
129.03
77.47
77.15
76.84
15.65
Cl
N
N
Cl
C5H4Cl2N2
Mol. Wt.: 163.00
BRUKER
NAME wenkun110831-2-C
EXPNO 1
PROCNO 1
Date_ 20110831
Time 14.53
INSTRUM spect
PROBHD 5 mm PABBO BB-
PULPROG zgpg30
TD 65536
SOLVENT CDCl3
NS 262
DS 4
SWH 24038.461 Hz
FIDRES 0.366798 Hz
AQ 1.3631988 sec
RG 456
DW 20.800 usec
DE 6.50 usec
TE 298.9 K
D1 2.00000000 sec
D11 0.03000000 sec
TD0 1
======= CHANNEL f1 =======
NUC1 13C
P1 10.10 usec
PL1 -3.00 dB
PL1W 64.15196228 W
SFO1 100.6228298 MHz
======= CHANNEL f2 =======
CPDPRG2 waltz16
NUC2 1H
PCPD2 80.00 usec
PL2 -4.00 dB
PL12 11.88 dB
PL13 12.00 dB
PL2W 20.19063568 W
PL12W 0.52137470 W
PL13W 0.50716585 W
SFO2 400.1316005 MHz
SI 32768
SF 100.6127690 MHz
WDW EM
SSB 0
LB 1.00 Hz
GB 0
PC 1.40
170
160
150
140
130
120
110
100
90
80
70
60
50
40
30
20
ppm

Compound 3b

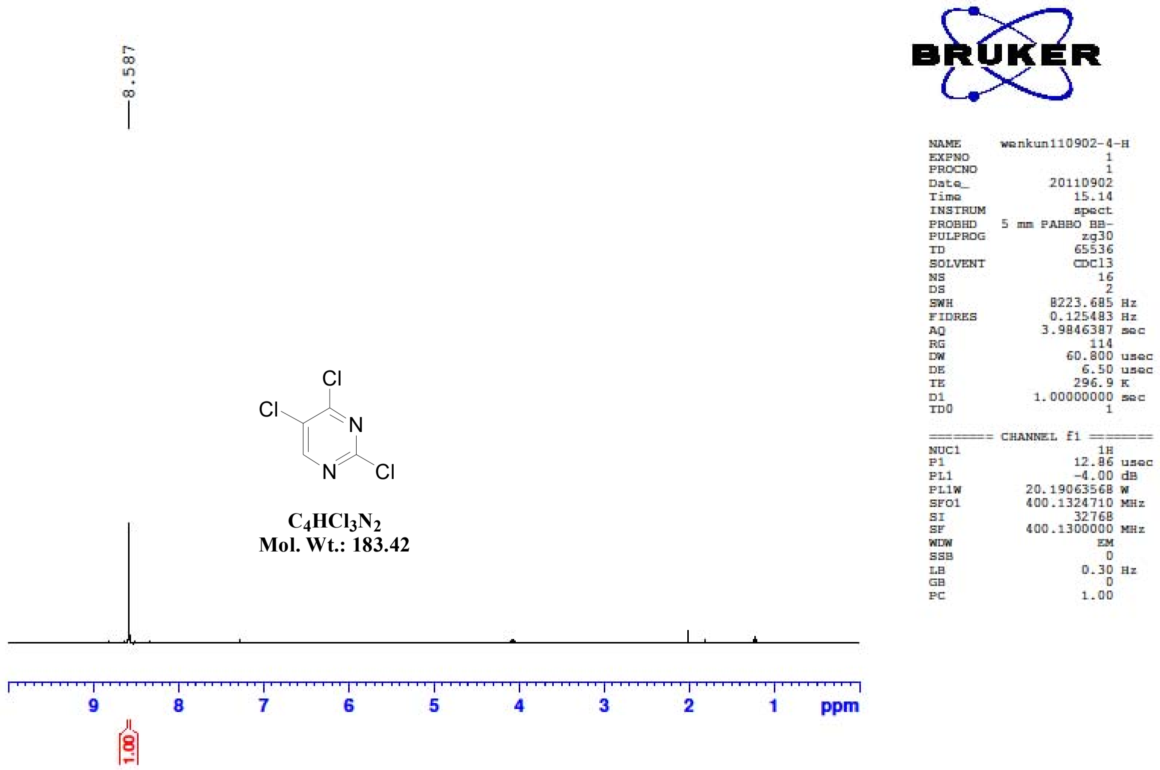
8.587
Cl
Cl
N
N
Cl
C4HCl3N2
Mol. Wt.: 183.42
9 8 7 6 5 4 3 2 1 ppm
1.00
BRUKER
NAME wenkun110902-4-H
EXPNO 1
PROCNO 1
Date_ 20110902
Time 15.14
INSTRUM spect
PROBHD 5 mm PABBO BB-
PULPROG zg30
TD 65536
SOLVENT CDCl3
NS 16
DS 2
SWH 8223.685 Hz
FIDRES 0.125483 Hz
AQ 3.9846387 sec
RG 114
DW 60.800 usec
DE 6.50 usec
TE 296.9 K
D1 1.00000000 sec
TD0 1
======== CHANNEL f1 ========
NUC1 1H
P1 12.86 usec
PL1 -4.00 dB
PL1W 20.19063568 W
SFO1 400.1324710 MHz
SI 32768
SF 400.1300000 MHz
WDW EM
SSB 0
LB 0.30 Hz
GB 0
PC 1.00

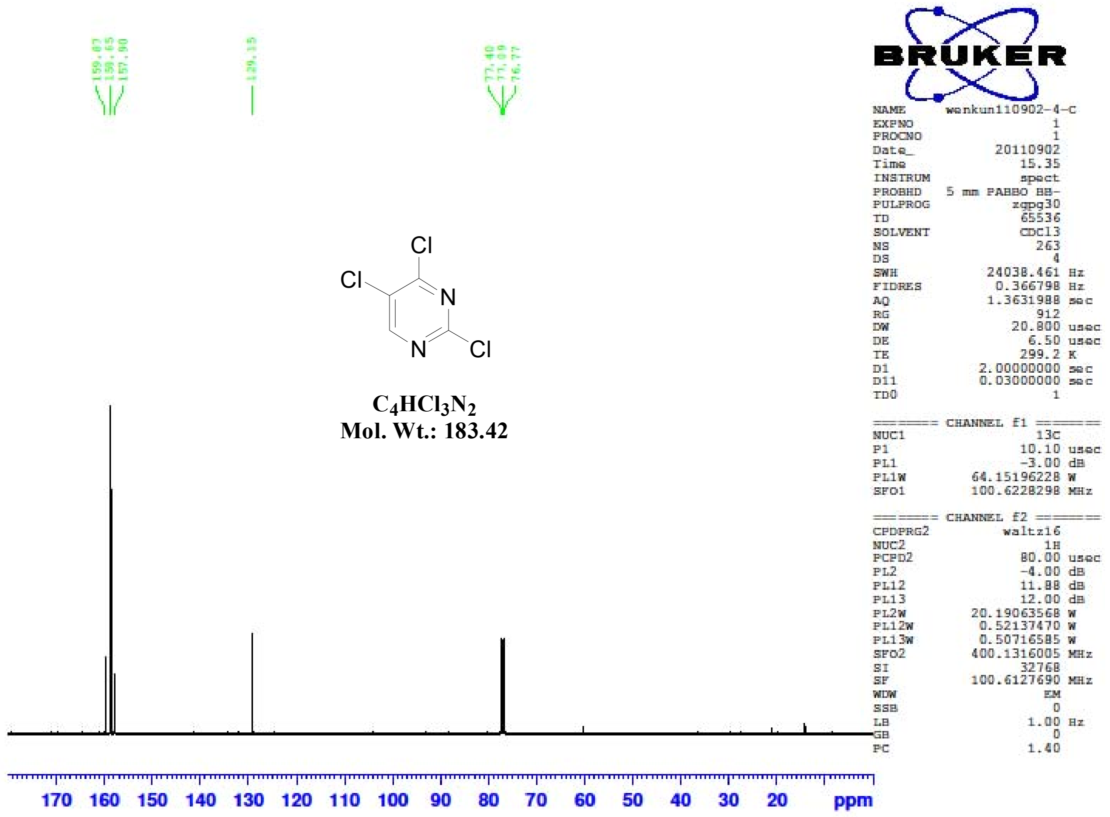
159.67
158.65
157.90
129.15
77.40
77.09
76.77
Cl
Cl
N
N
Cl
C4HCl3N2
Mol. Wt.: 183.42
170 160 150 140 130 120 110 100 90 80 70 60 50 40 30 20 ppm
BRUKER
NAME wenkun110902-4-C
EXPNO 1
PROCNO 1
Date_ 20110902
Time 15.35
INSTRUM spect
PROBHD 5 mm PABBO BB-
PULPROG zgpg30
TD 65536
SOLVENT CDCl3
NS 263
DS 4
SWH 24038.461 Hz
FIDRES 0.366798 Hz
AQ 1.3631988 sec
RG 912
DW 20.800 usec
DE 6.50 usec
TE 299.2 K
D1 2.00000000 sec
D11 0.03000000 sec
TD0 1
======== CHANNEL f1 ========
NUC1 13C
P1 10.10 usec
PL1 -3.00 dB
PL1W 64.15196228 W
SFO1 100.6228298 MHz
======== CHANNEL f2 ========
CPDPRG2 waltz16
NUC2 1H
PCPD2 80.00 usec
PL2 -4.00 dB
PL12 11.88 dB
PL13 12.00 dB
PL2W 20.19063568 W
PL12W 0.52137470 W
PL13W 0.50716585 W
SFO2 400.1316005 MHz
SI 32768
SF 100.6127690 MHz
WDW EM
SSB 0
LB 1.00 Hz
GB 0
PC 1.40

Compound 4b

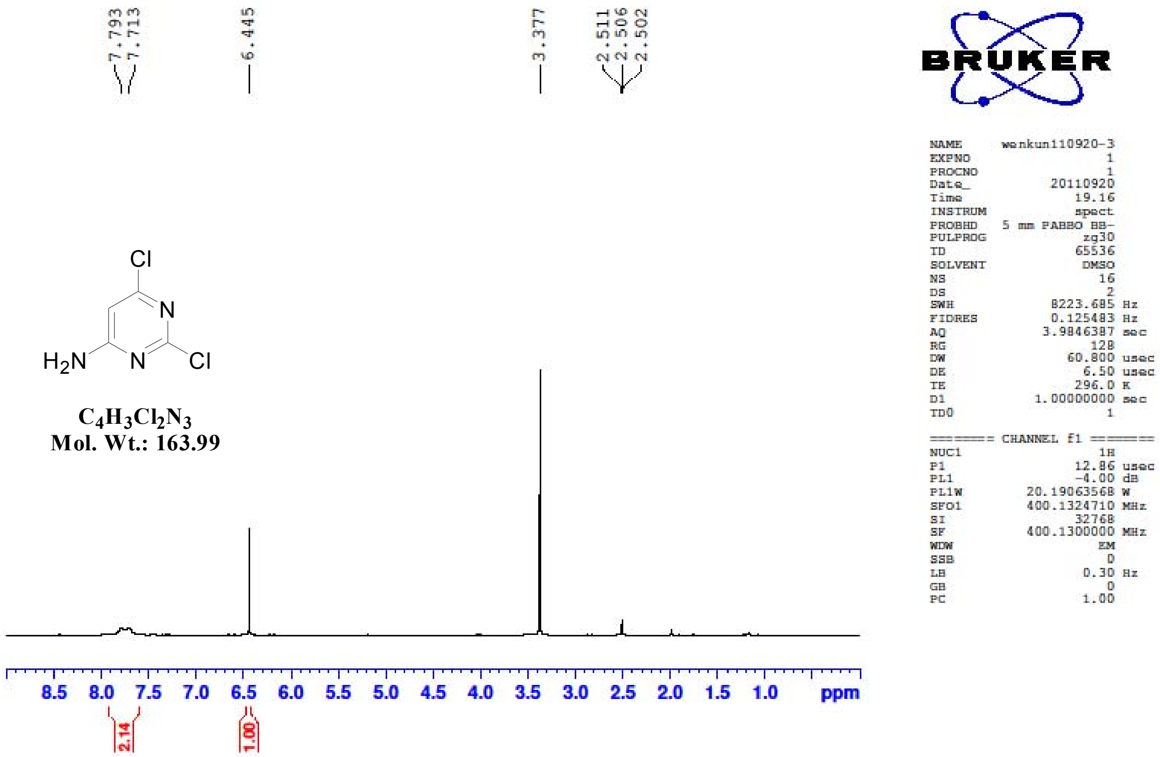
7.793
7.713
6.445
3.377
2.511
2.506
2.502
BRUKER
NAME wenkun110920-3
EXPNO 1
PROCNO 1
Date_ 20110920
Time 19.16
INSTRUM spect
PROBHD 5 mm PABBO BB-
PULPROG zg30
TD 65536
SOLVENT DMSO
NS 16
DS 2
SWH 8223.685 Hz
FIDRES 0.125483 Hz
AQ 3.9846387 sec
RG 128
DW 60.800 usec
DE 6.50 usec
TE 296.0 K
D1 1.00000000 sec
TD0 1
======== CHANNEL f1 ========
NUC1 1H
P1 12.86 usec
PL1 -4.00 dB
PL1W 20.19063568 W
SFO1 400.1324710 MHz
SI 32768
SF 400.1300000 MHz
WDW EM
SSB 0
LB 0.30 Hz
GB 0
PC 1.00
Cl
N
H2N
N
Cl
C4H3Cl2N3
Mol. Wt.: 163.99
8.5 8.0 7.5 7.0 6.5 6.0 5.5 5.0 4.5 4.0 3.5 3.0 2.5 2.0 1.5 1.0 ppm
2.14
1.00

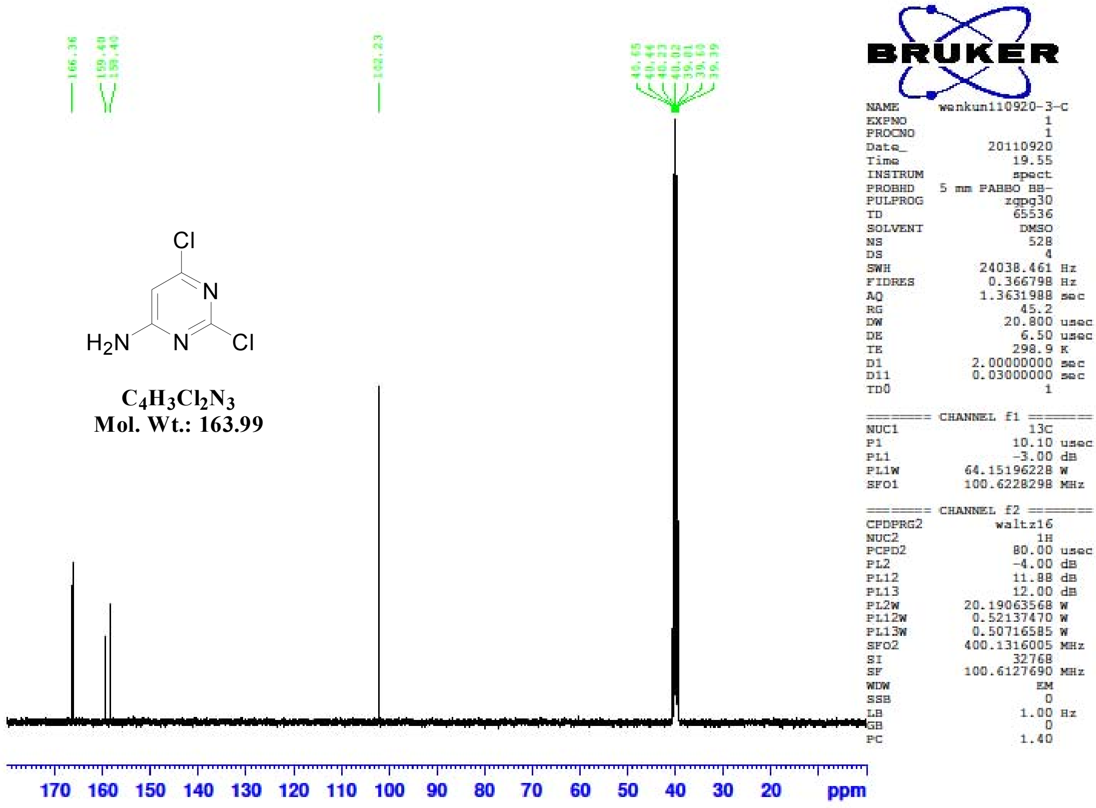
166.36
BRUKER
NAME wenkun110920-3-C
EXPNO 1
PROCNO 1
Date_ 20110920
Time 19.55
INSTRUM spect
PROBHD 5 mm PABBO BB-
PULPROG zgpg30
TD 65536
SOLVENT DMSO
NS 528
DS 4
SWH 24038.461 Hz
FIDRES 0.366798 Hz
AQ 1.3631988 sec
RG 45.2
DW 20.800 usec
DE 6.50 usec
TE 298.9 K
D1 2.00000000 sec
D11 0.03000000 sec
TD0 1
======== CHANNEL f1 ========
NUC1 13C
P1 10.10 usec
PL1 -3.00 dB
PL1W 64.15196228 W
SFO1 100.6228298 MHz
======== CHANNEL f2 ========
CPDPRG2 waltz16
NUC2 1H
PCPD2 80.00 usec
PL2 -4.00 dB
PL12 11.88 dB
PL13 12.00 dB
PL2W 20.19063568 W
PL12W 0.52137470 W
PL13W 0.50716585 W
SFO2 400.1316005 MHz
SI 32768
SF 100.6127690 MHz
WDW EM
SSB 0
LB 1.00 Hz
GB 0
PC 1.40
Cl
N
H2N
N
Cl
C4H3Cl2N3
Mol. Wt.: 163.99
170 160 150 140 130 120 110 100 90 80 70 60 50 40 30 20 ppm

Compound 5b

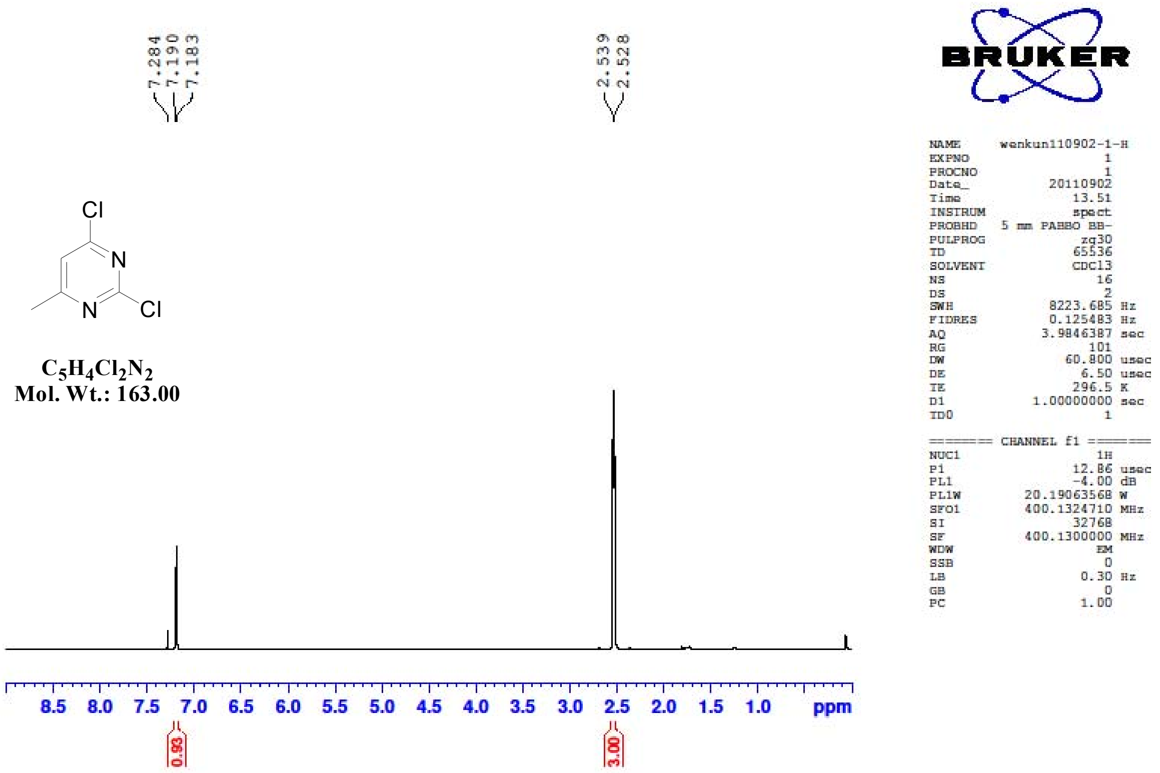
7.284
7.190
7.183
2.539
2.528
Cl
N
N
Cl
$C_5H_4Cl_2N_2$
Mol. Wt.: 163.00
0.93
3.00
8.5 8.0 7.5 7.0 6.5 6.0 5.5 5.0 4.5 4.0 3.5 3.0 2.5 2.0 1.5 1.0 ppm
BRUKER
NAME wenkun110902-1-H
EXPNO 1
PROCNO 1
Date_ 20110902
Time 13.51
INSTRUM spect
PROBHD 5 mm PABBO BB-
PULPROG zg30
TD 65536
SOLVENT CDCl3
NS 16
DS 2
SWH 8223.685 Hz
FIDRES 0.125483 Hz
AQ 3.9846387 sec
RG 101
DW 60.800 usec
DE 6.50 usec
TE 296.5 K
D1 1.00000000 sec
TD0 1
====== CHANNEL f1 ======
NUC1 1H
P1 12.86 usec
PL1 -4.00 dB
PL1W 20.19063568 W
SFO1 400.1324710 MHz
SI 32768
SF 400.1300000 MHz
WDW EM
SSB 0
LB 0.30 Hz
GB 0
PC 1.00

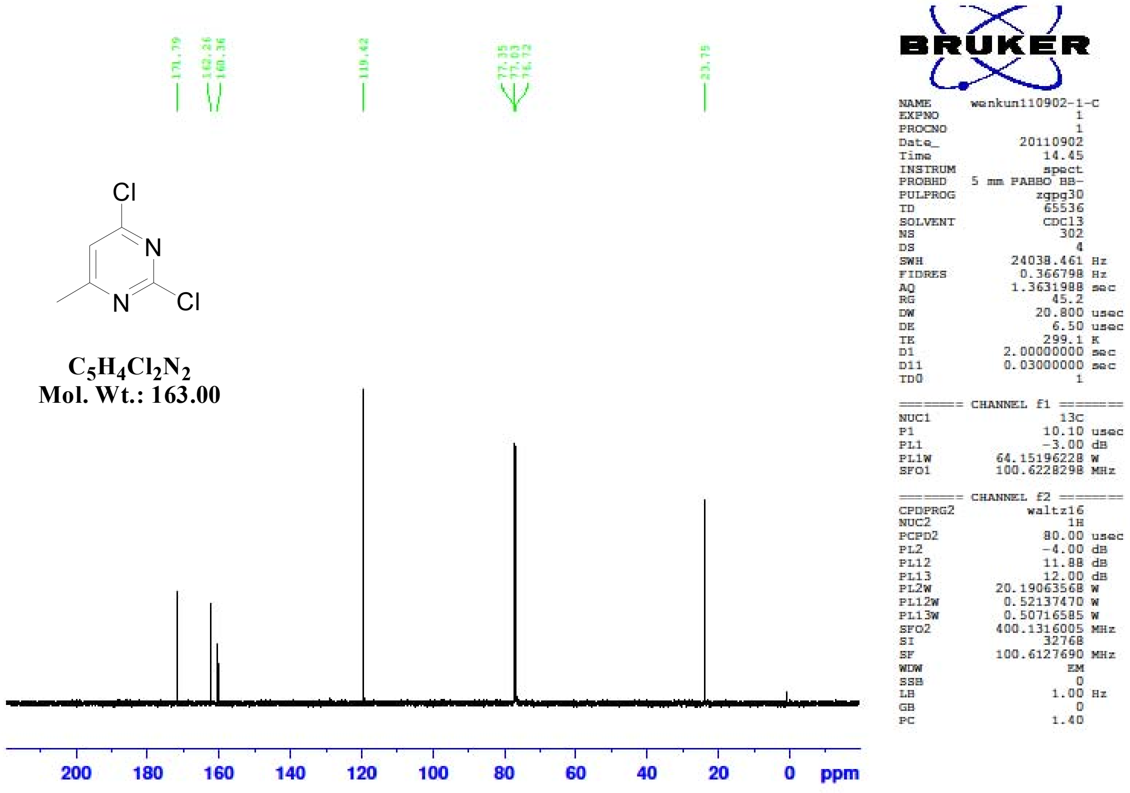
171.79
162.26
160.36
119.42
77.35
77.03
76.72
23.75
Cl
N
N
Cl
$C_5H_4Cl_2N_2$
Mol. Wt.: 163.00
200 180 160 140 120 100 80 60 40 20 0 ppm
BRUKER
NAME wenkun110902-1-C
EXPNO 1
PROCNO 1
Date_ 20110902
Time 14.45
INSTRUM spect
PROBHD 5 mm PABBO BB-
PULPROG zgpg30
TD 65536
SOLVENT CDCl3
NS 302
DS 4
SWH 24038.461 Hz
FIDRES 0.366798 Hz
AQ 1.3631988 sec
RG 45.2
DW 20.800 usec
DE 6.50 usec
TE 299.1 K
D1 2.00000000 sec
D11 0.03000000 sec
TD0 1
====== CHANNEL f1 ======
NUC1 13C
P1 10.10 usec
PL1 -3.00 dB
PL1W 64.15196228 W
SFO1 100.6228298 MHz
====== CHANNEL f2 ======
CPDPRG2 waltz16
NUC2 1H
PCPD2 80.00 usec
PL2 -4.00 dB
PL12 11.88 dB
PL13 12.00 dB
PL2W 20.19063568 W
PL12W 0.52137470 W
PL13W 0.50716585 W
SFO2 400.1316005 MHz
SI 32768
SF 100.6127690 MHz
WDW EM
SSB 0
LB 1.00 Hz
GB 0
PC 1.40

Compound 6b

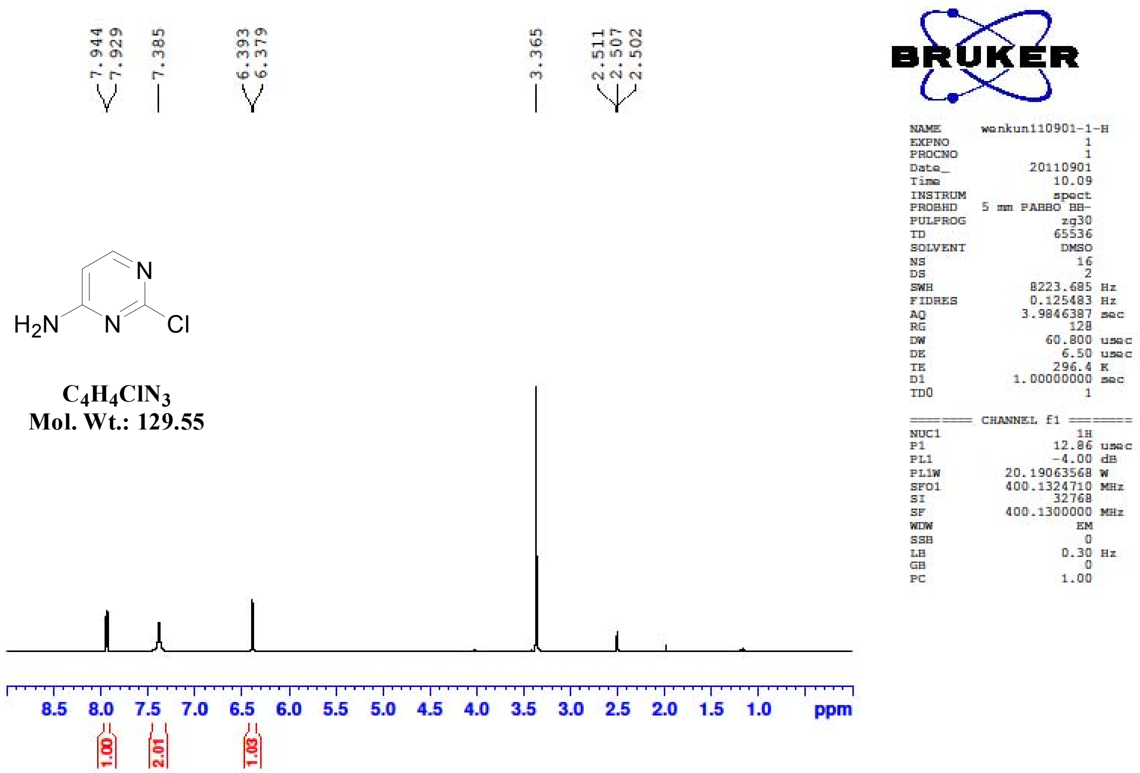
7.944
7.929
7.385
6.393
6.379
3.365
2.511
2.507
2.502
BRUKER
NAME wenkun110901-1-H
EXPNO 1
PROCNO 1
Date_ 20110901
Time 10.09
INSTRUM spect
PROBHD 5 mm PABBO BB-
PULPROG zg30
TD 65536
SOLVENT DMSO
NS 16
DS 2
SWH 8223.685 Hz
FIDRES 0.125483 Hz
AQ 3.9846387 sec
RG 128
DW 60.800 usec
DE 6.50 usec
TE 296.4 K
D1 1.00000000 sec
TD0 1
======= CHANNEL f1 =======
NUC1 1H
P1 12.86 usec
PL1 -4.00 dB
PL1W 20.19063568 W
SFO1 400.1324710 MHz
SI 32768
SF 400.1300000 MHz
WDW EM
SSB 0
LB 0.30 Hz
GB 0
PC 1.00
H2N
N
N
Cl
$C_4H_4ClN_3$
Mol. Wt.: 129.55
8.5 8.0 7.5 7.0 6.5 6.0 5.5 5.0 4.5 4.0 3.5 3.0 2.5 2.0 1.5 1.0 ppm
1.00
2.01
1.03

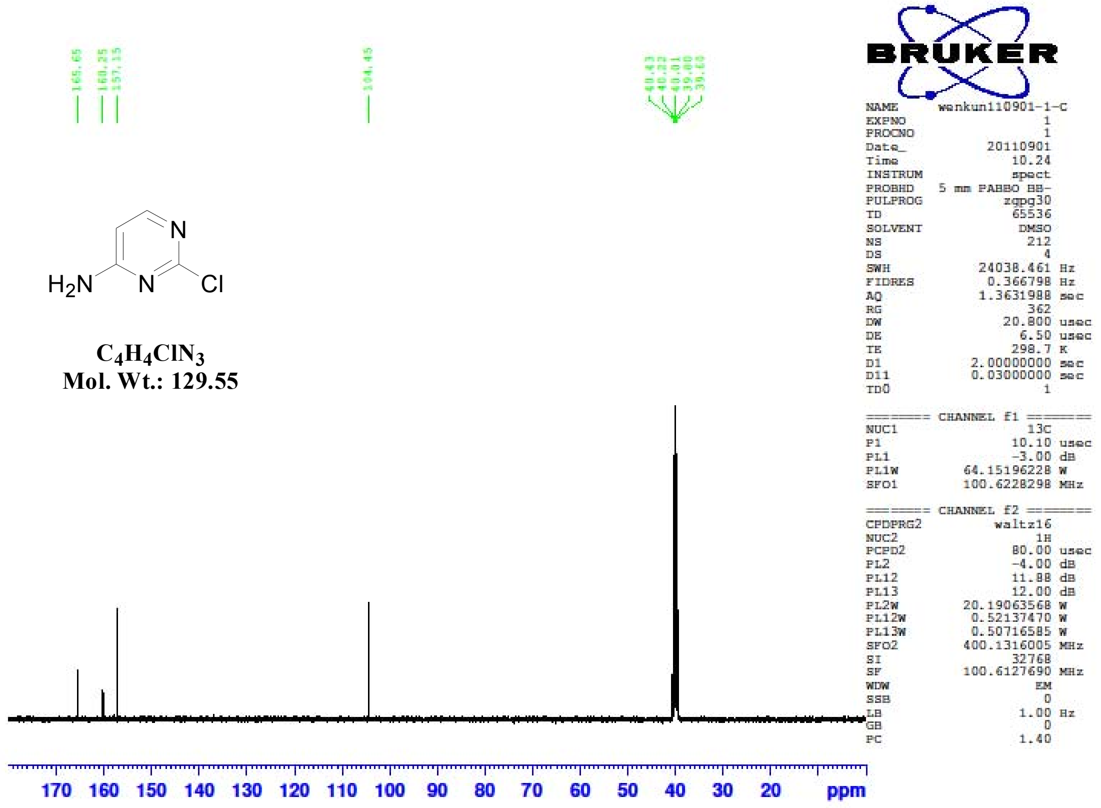
165.65
160.25
157.15
104.45
BRUKER
NAME wenkun110901-1-C
EXPNO 1
PROCNO 1
Date_ 20110901
Time 10.24
INSTRUM spect
PROBHD 5 mm PABBO BB-
PULPROG zgpg30
TD 65536
SOLVENT DMSO
NS 212
DS 4
SWH 24038.461 Hz
FIDRES 0.366798 Hz
AQ 1.3631988 sec
RG 362
DW 20.800 usec
DE 6.50 usec
TE 298.7 K
D1 2.00000000 sec
D11 0.03000000 sec
TD0 1
======= CHANNEL f1 =======
NUC1 13C
P1 10.10 usec
PL1 -3.00 dB
PL1W 64.15196228 W
SFO1 100.6228298 MHz
======= CHANNEL f2 =======
CPDPRG2 waltz16
NUC2 1H
PCPD2 80.00 usec
PL2 -4.00 dB
PL12 11.88 dB
PL13 12.00 dB
PL2W 20.19063568 W
PL12W 0.52137470 W
PL13W 0.50716585 W
SFO2 400.1316005 MHz
SI 32768
SF 100.6127690 MHz
WDW EM
SSB 0
LB 1.00 Hz
GB 0
PC 1.40
H2N
N
N
Cl
$C_4H_4ClN_3$
Mol. Wt.: 129.55
170 160 150 140 130 120 110 100 90 80 70 60 50 40 30 20 ppm

Compound 7b

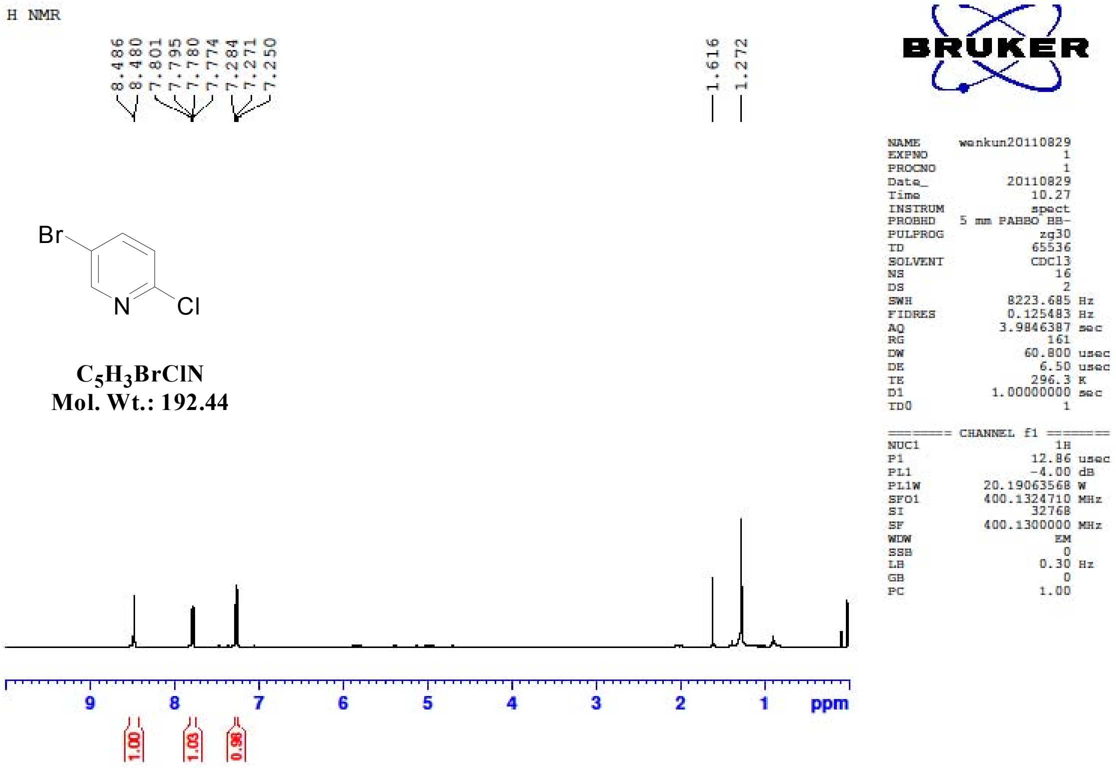

H NMR
8.486
8.480
7.801
7.795
7.780
7.774
7.284
7.271
7.250
1.616
1.272
Br
N
Cl
C_5H_3BrClN
Mol. Wt.: 192.44
BRUKER
ppm
1.00
1.03
0.98

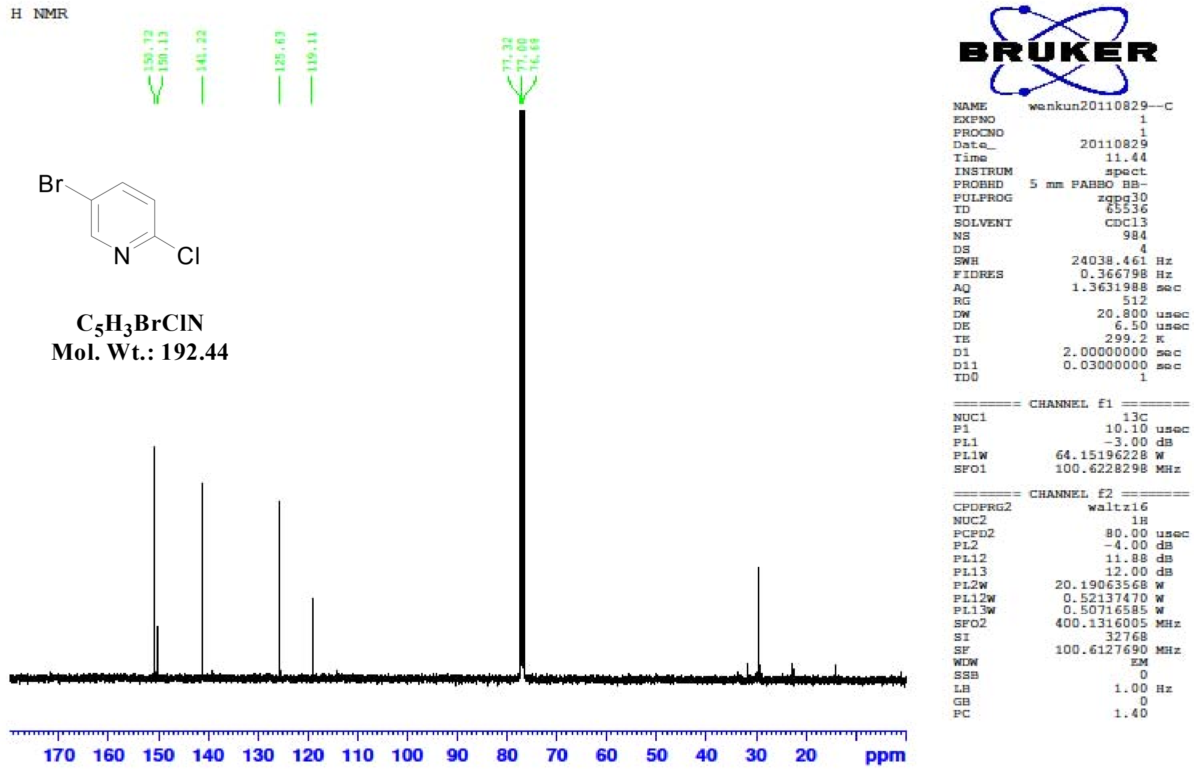

H NMR
Br
N
Cl
C_5H_3BrClN
Mol. Wt.: 192.44
BRUKER
ppm

Compound 8b

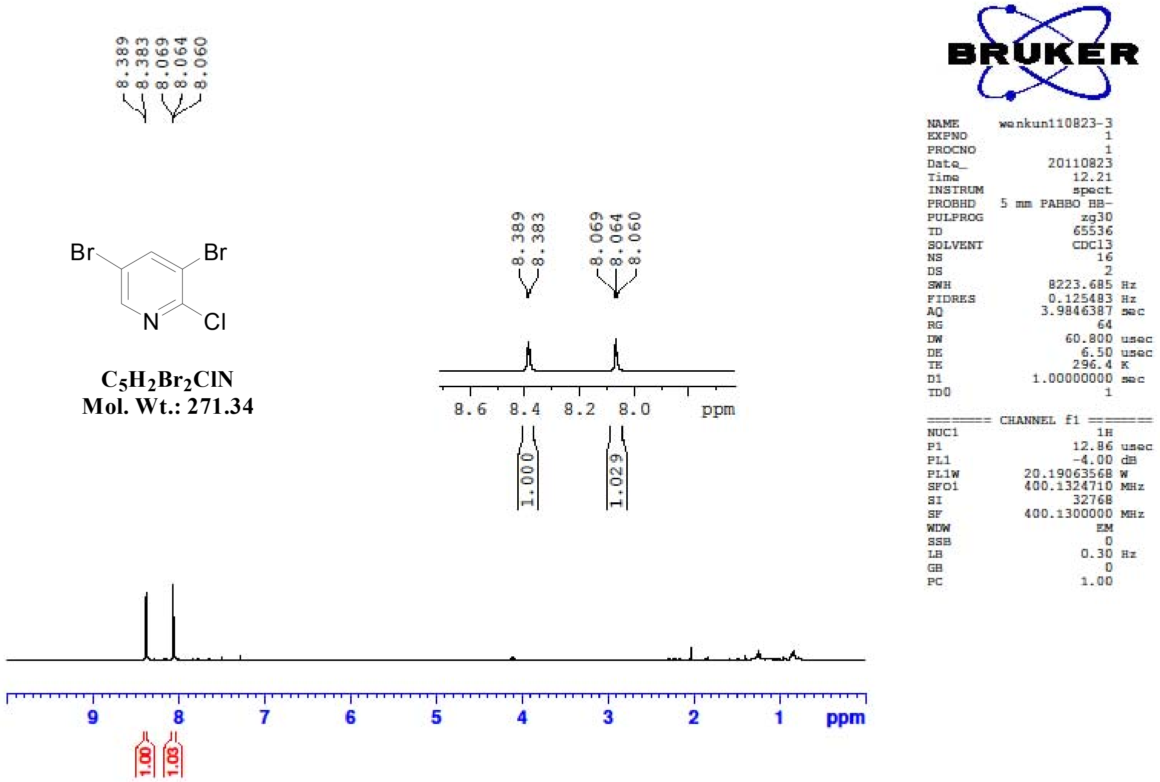
8.389
8.383
8.069
8.064
8.060
Br
Br
N
Cl
$C_5H_2Br_2ClN$
Mol. Wt.: 271.34
8.6 8.4 8.2 8.0 ppm
1.000
1.029
9 8 7 6 5 4 3 2 1 ppm
1.00
1.03
BRUKER
NAME wenkun110823-3
EXPNO 1
PROCNO 1
Date_ 20110823
Time 12.21
INSTRUM spect
PROBHD 5 mm PABBO BB-
PULPROG zg30
TD 65536
SOLVENT CDCl3
NS 16
DS 2
SWH 8223.685 Hz
FIDRES 0.125483 Hz
AQ 3.9846387 sec
RG 64
DW 60.800 usec
DE 6.50 usec
TE 296.4 K
D1 1.00000000 sec
TD0 1
======== CHANNEL f1 ========
NUC1 1H
P1 12.86 usec
PL1 -4.00 dB
PL1W 20.19063568 W
SFO1 400.1324710 MHz
SI 32768
SF 400.1300000 MHz
WDW EM
SSB 0
LB 0.30 Hz
GB 0
PC 1.00

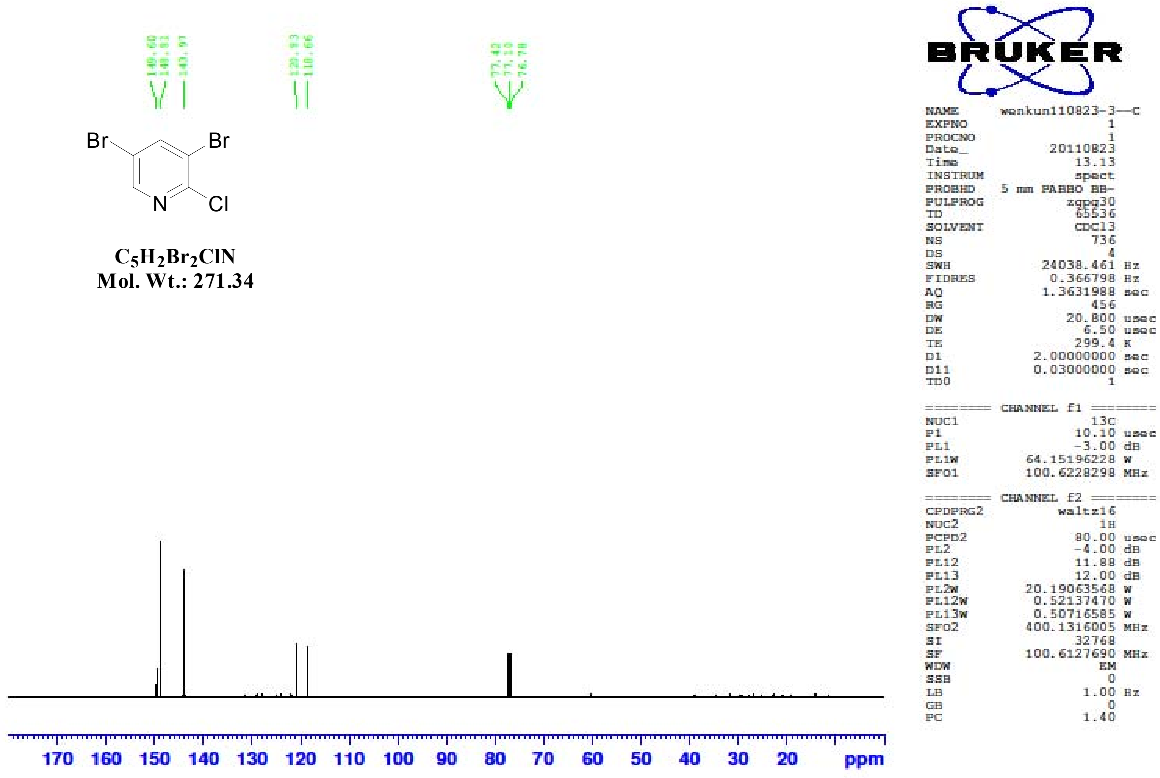
149.60
148.81
143.97
120.93
118.66
77.42
77.10
76.78
Br
Br
N
Cl
$C_5H_2Br_2ClN$
Mol. Wt.: 271.34
170 160 150 140 130 120 110 100 90 80 70 60 50 40 30 20 ppm
BRUKER
NAME wenkun110823-3—C
EXPNO 1
PROCNO 1
Date_ 20110823
Time 13.13
INSTRUM spect
PROBHD 5 mm PABBO BB-
PULPROG zgpg30
TD 65536
SOLVENT CDCl3
NS 736
DS 4
SWH 24038.461 Hz
FIDRES 0.366798 Hz
AQ 1.3631988 sec
RG 456
DW 20.800 usec
DE 6.50 usec
TE 299.4 K
D1 2.00000000 sec
D11 0.03000000 sec
TD0 1
======== CHANNEL f1 ========
NUC1 13C
P1 10.10 usec
PL1 -3.00 dB
PL1W 64.15196228 W
SFO1 100.6228298 MHz
======== CHANNEL f2 ========
CPDPRG2 waltz16
NUC2 1H
PCPD2 80.00 usec
PL2 -4.00 dB
PL12 11.88 dB
PL13 12.00 dB
PL2W 20.19063568 W
PL12W 0.52137470 W
PL13W 0.50716585 W
SFO2 400.1316005 MHz
SI 32768
SF 100.6127690 MHz
WDW EM
SSB 0
LB 1.00 Hz
GB 0
PC 1.40

Compound 9b

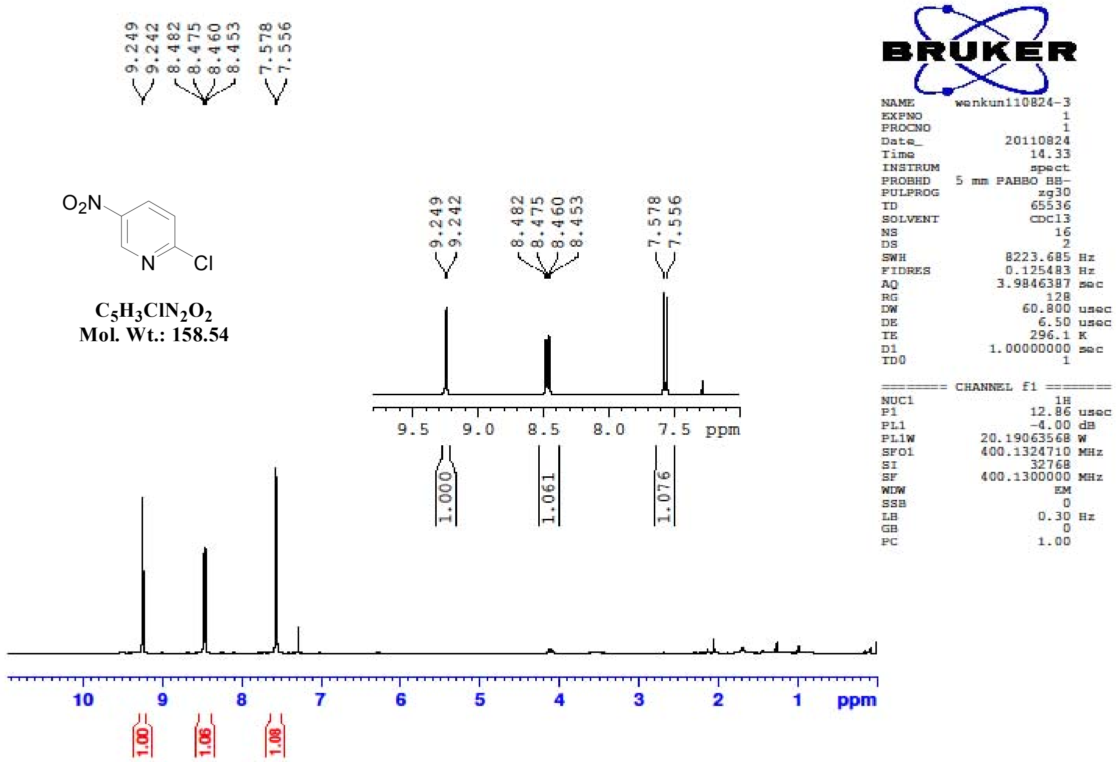
9.249
9.242
8.482
8.475
8.460
8.453
7.578
7.556
O2N
N
Cl
C5H3ClN2O2
Mol. Wt.: 158.54
1.000
1.061
1.076
9.5 9.0 8.5 8.0 7.5 ppm
BRUKER
NAME wenkun110824-3
EXPNO 1
PROCNO 1
Date_ 20110824
Time 14.33
INSTRUM spect
PROBHD 5 mm PABBO BB-
PULPROG zg30
TD 65536
SOLVENT CDCl3
NS 16
DS 2
SWH 8223.685 Hz
FIDRES 0.125483 Hz
AQ 3.9846387 sec
RG 128
DW 60.800 usec
DE 6.50 usec
TE 296.1 K
D1 1.00000000 sec
TD0 1
======= CHANNEL f1 =======
NUC1 1H
P1 12.86 usec
PL1 -4.00 dB
PL1W 20.19063568 W
SFO1 400.1324710 MHz
SI 32768
SF 400.1300000 MHz
WDW EM
SSB 0
LB 0.30 Hz
GB 0
PC 1.00
10 9 8 7 6 5 4 3 2 1 ppm
1.00
1.06
1.08

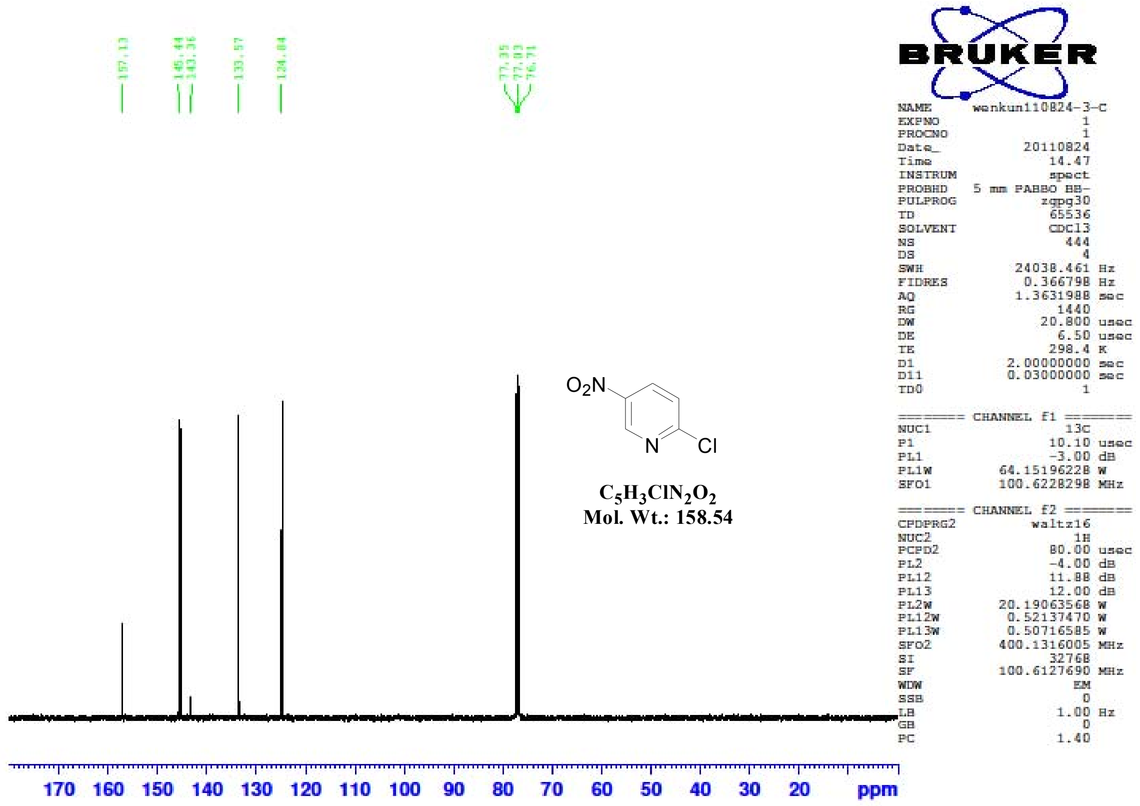
157.13
145.44
143.36
133.57
124.84
77.35
77.03
76.71
BRUKER
NAME wenkun110824-3-C
EXPNO 1
PROCNO 1
Date_ 20110824
Time 14.47
INSTRUM spect
PROBHD 5 mm PABBO BB-
PULPROG zgpg30
TD 65536
SOLVENT CDCl3
NS 444
DS 4
SWH 24038.461 Hz
FIDRES 0.366798 Hz
AQ 1.3631988 sec
RG 1440
DW 20.800 usec
DE 6.50 usec
TE 298.4 K
D1 2.00000000 sec
D11 0.03000000 sec
TD0 1
======= CHANNEL f1 =======
NUC1 13C
P1 10.10 usec
PL1 -3.00 dB
PL1W 64.15196228 W
SFO1 100.6228298 MHz
======= CHANNEL f2 =======
CPDPRG2 waltz16
NUC2 1H
PCPD2 80.00 usec
PL2 -4.00 dB
PL12 11.88 dB
PL13 12.00 dB
PL2W 20.19063568 W
PL12W 0.52137470 W
PL13W 0.50716585 W
SFO2 400.1316005 MHz
SI 32768
SF 100.6127690 MHz
WDW EM
SSB 0
LB 1.00 Hz
GB 0
PC 1.40
O2N
N
Cl
C5H3ClN2O2
Mol. Wt.: 158.54
170 160 150 140 130 120 110 100 90 80 70 60 50 40 30 20 ppm

Compound 10b

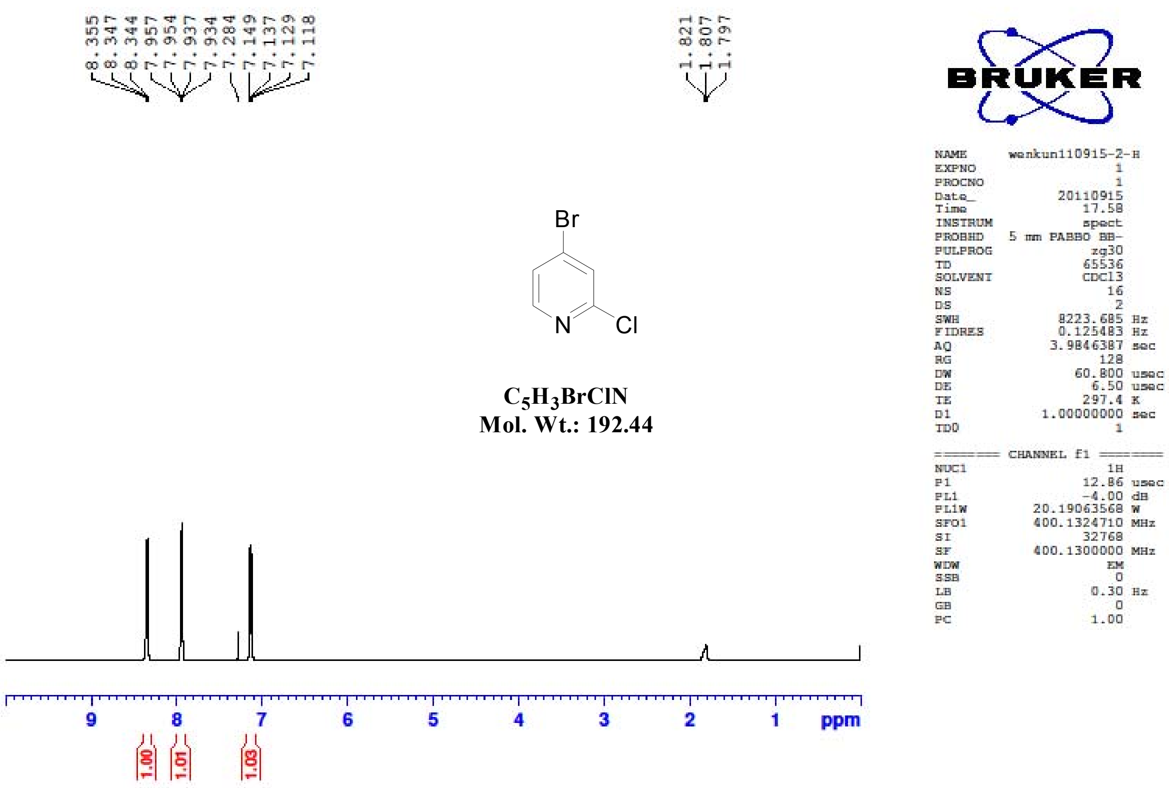

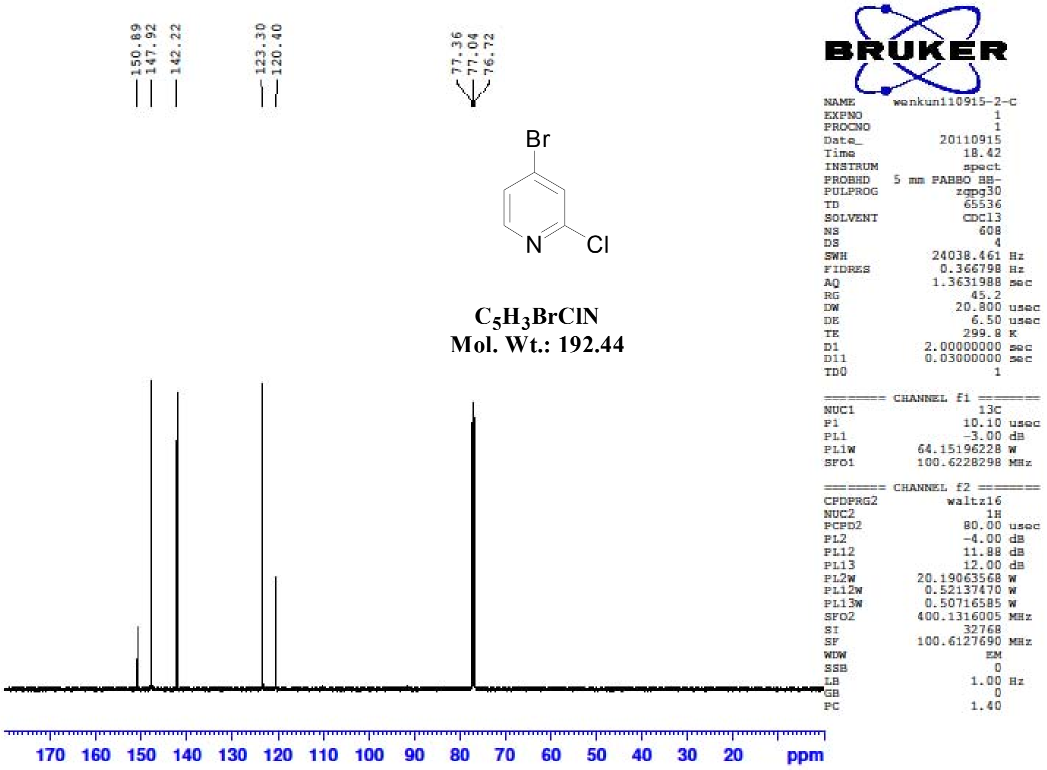

Compound 11b

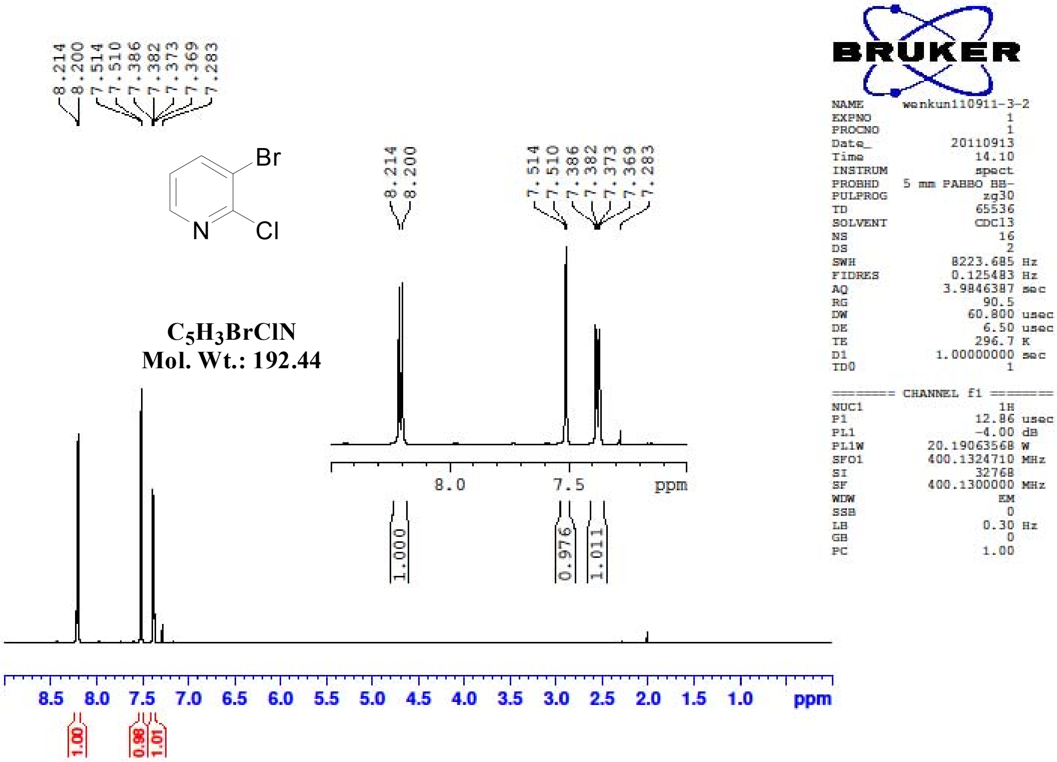

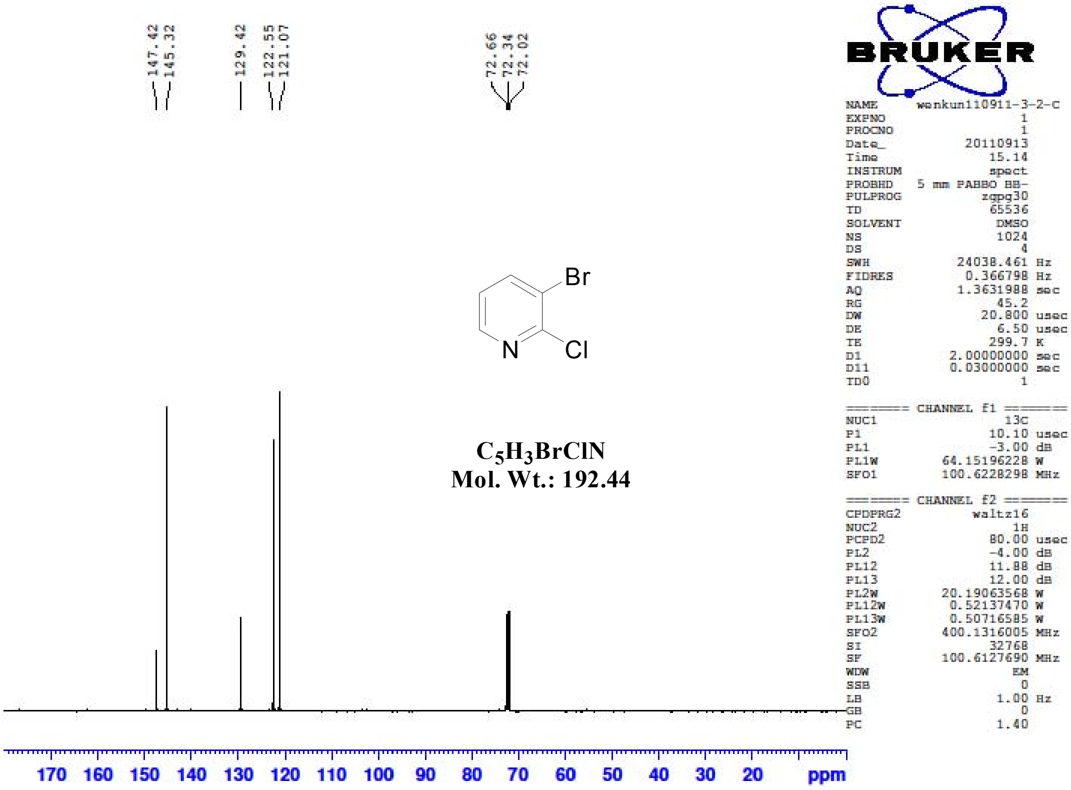

Compound 12b

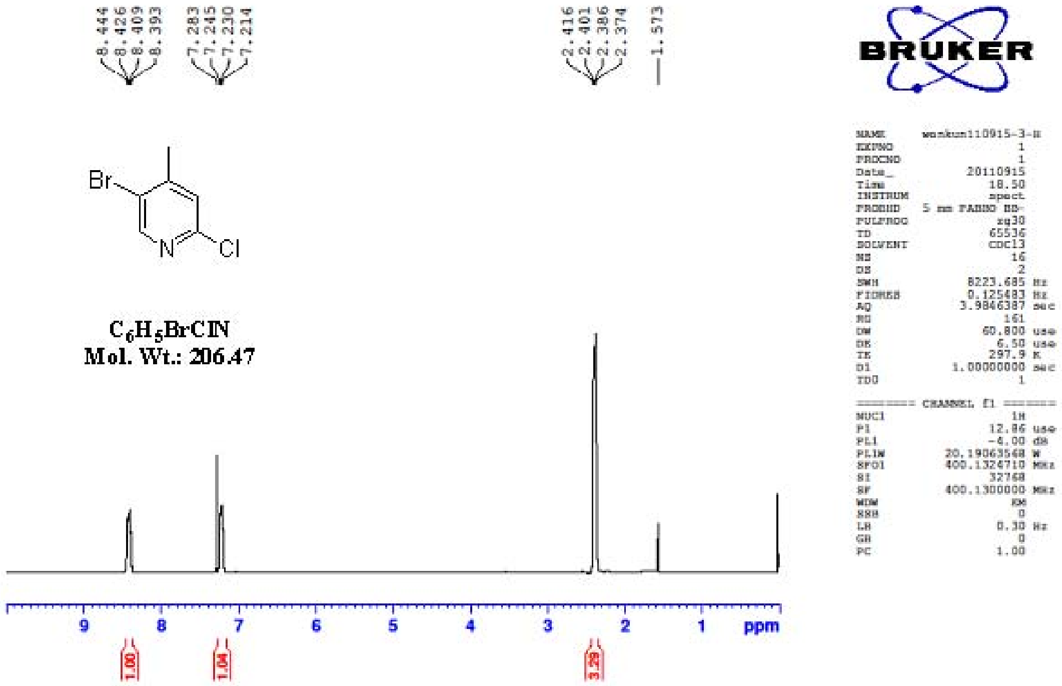

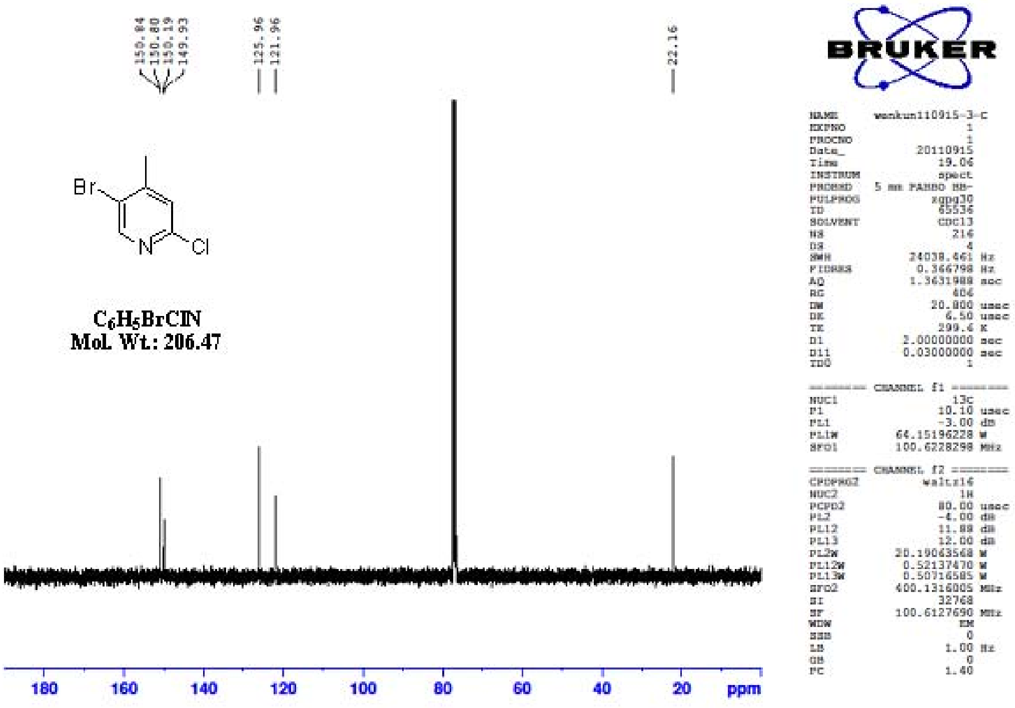

Compound 14b

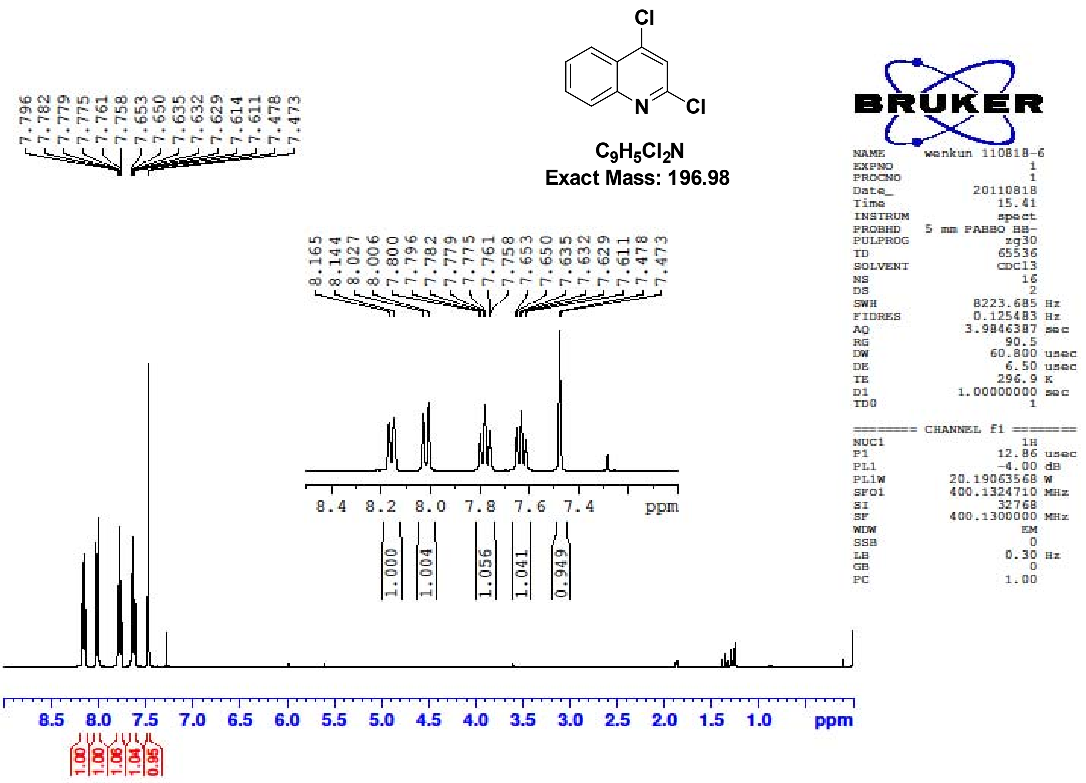

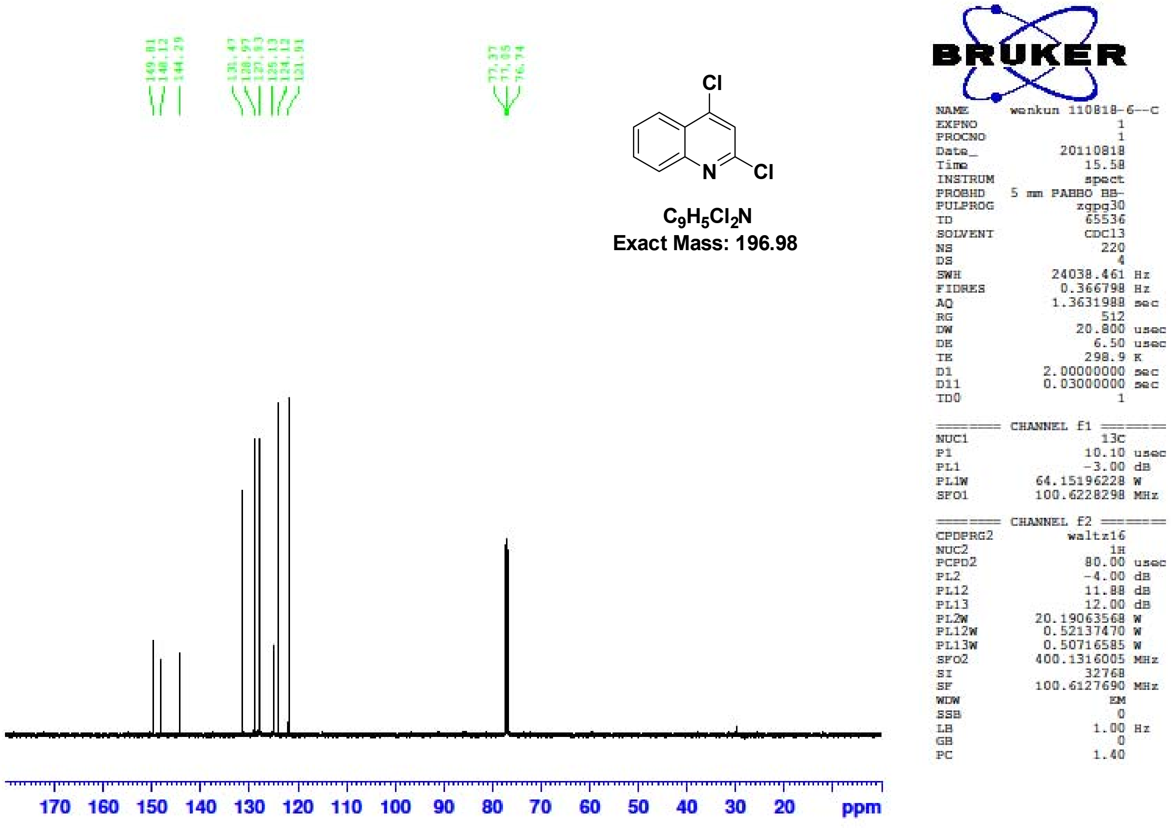

Compound 15b

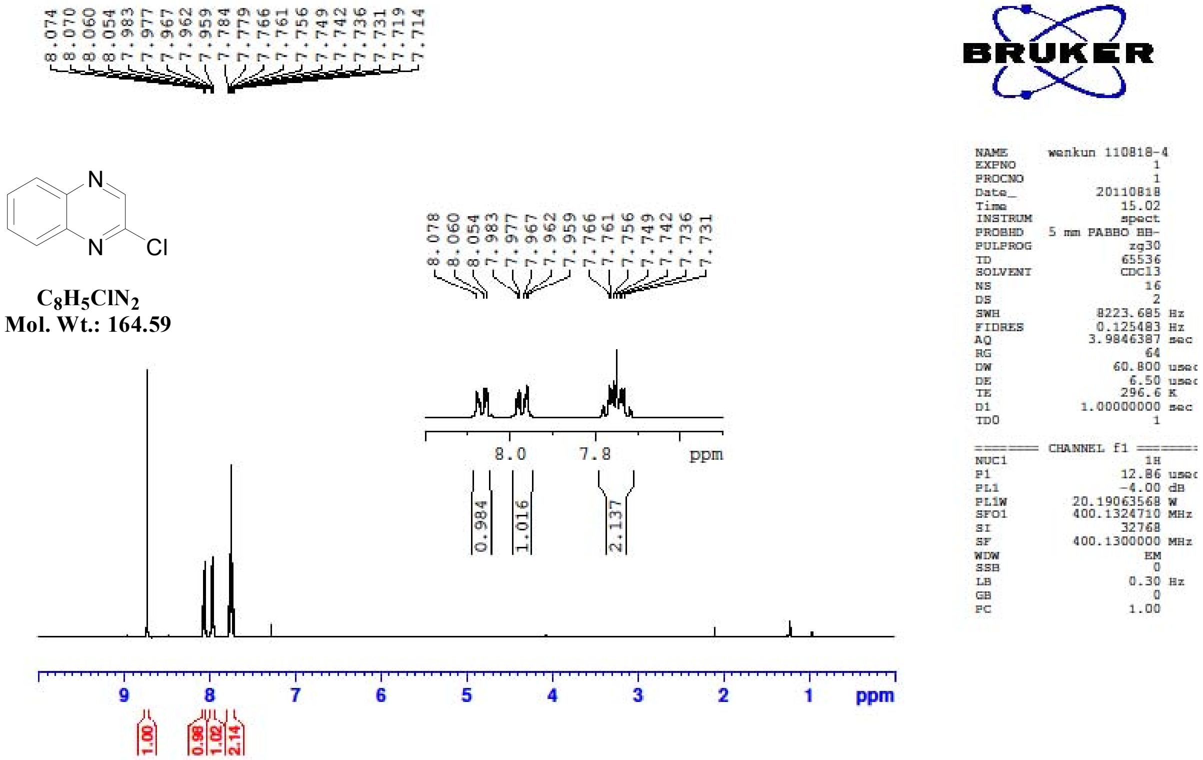

$C_8H_5ClN_2$
Mol. Wt.: 164.59

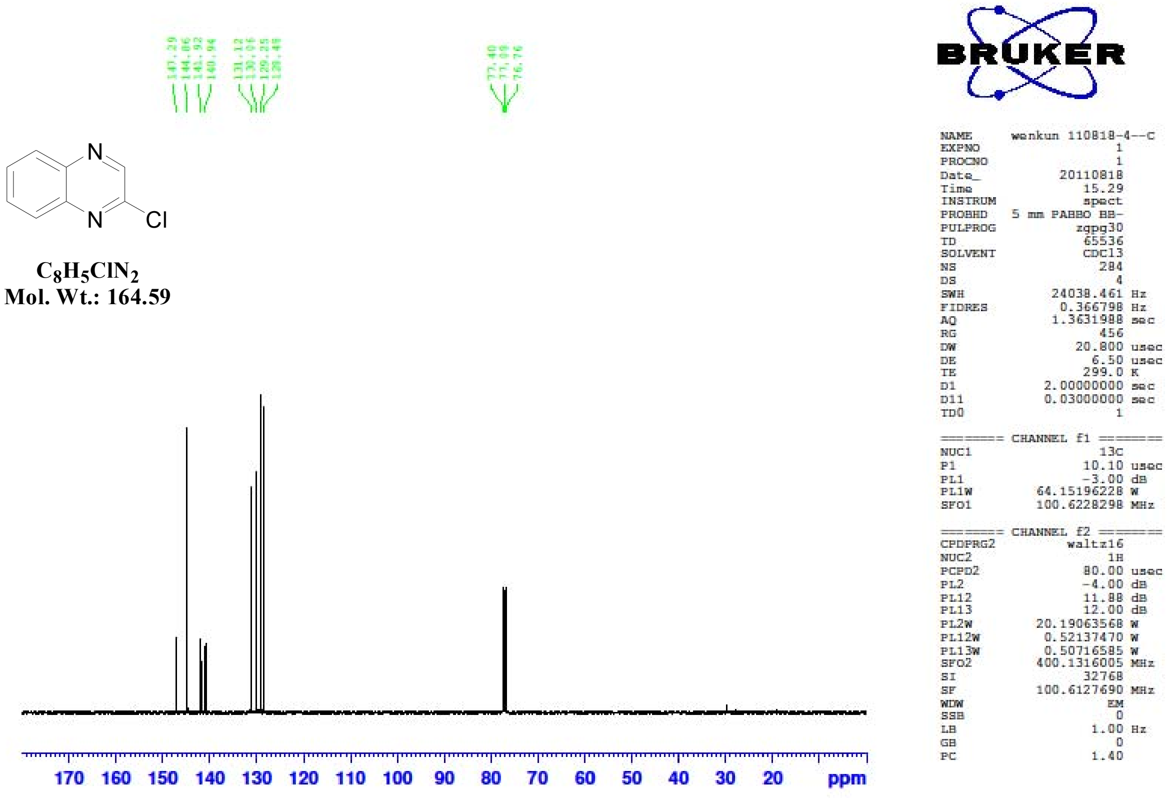

$C_8H_5ClN_2$
Mol. Wt.: 164.59

Compound 16b

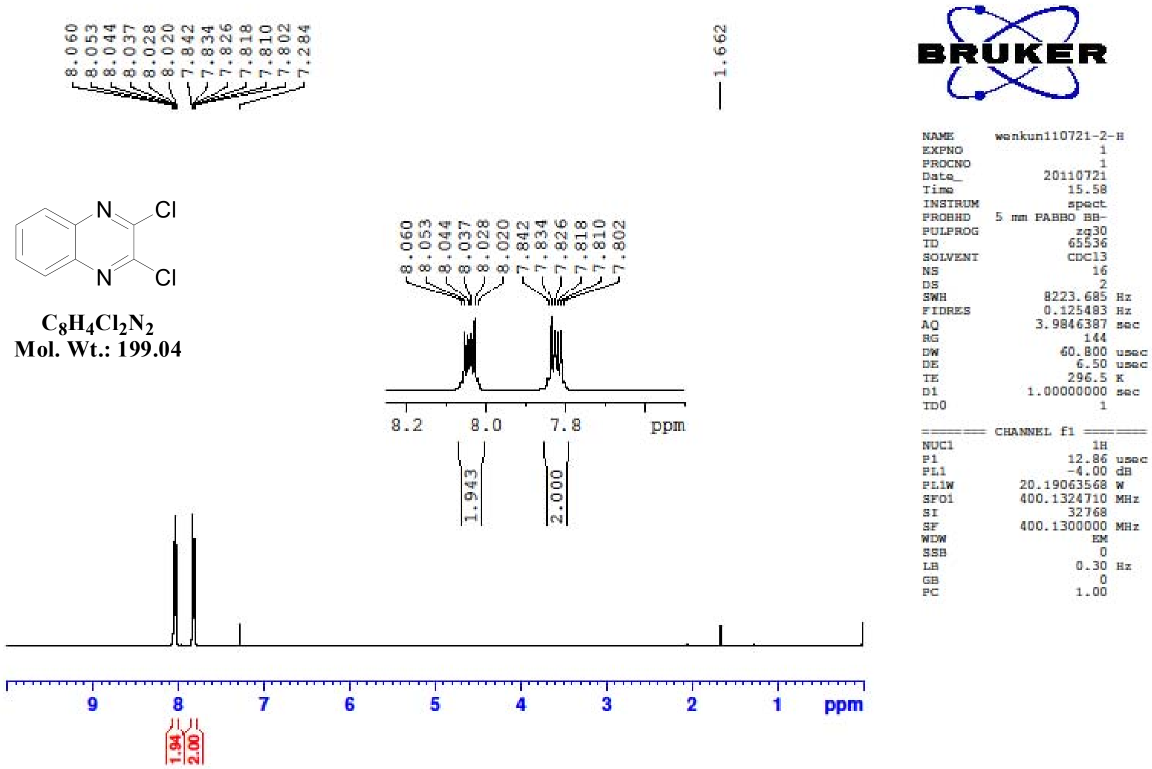

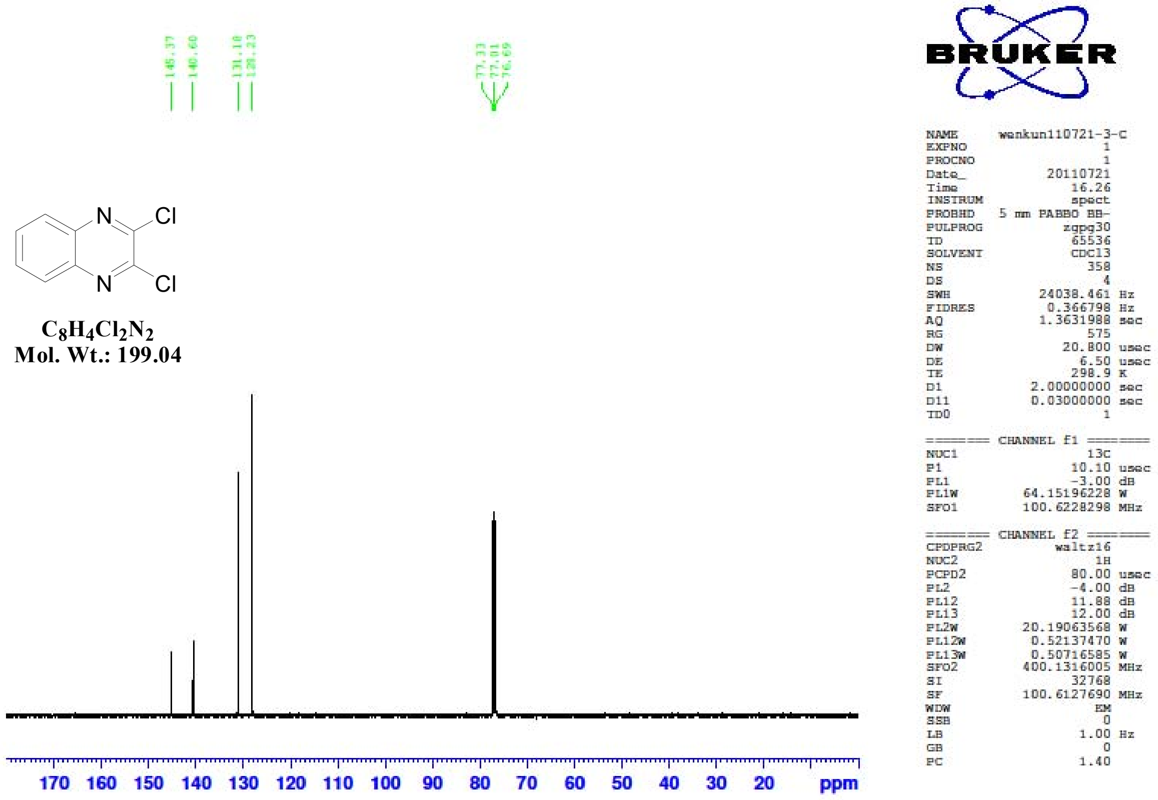

Compound 17b

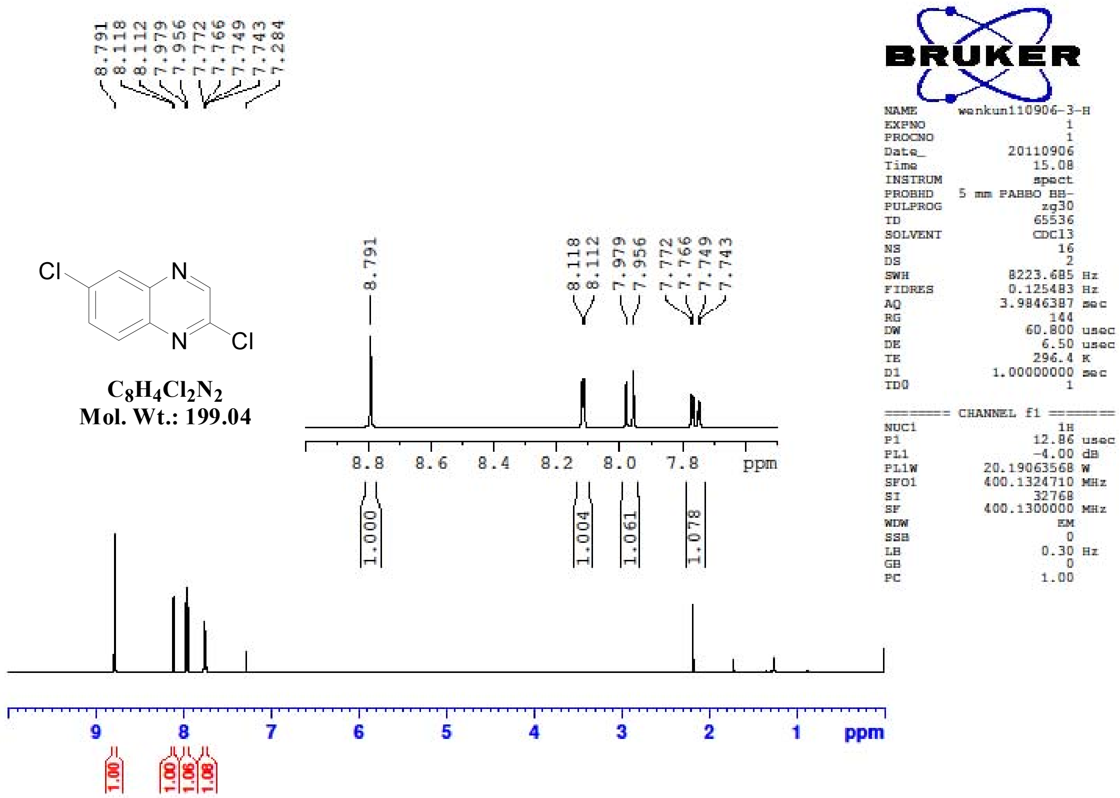

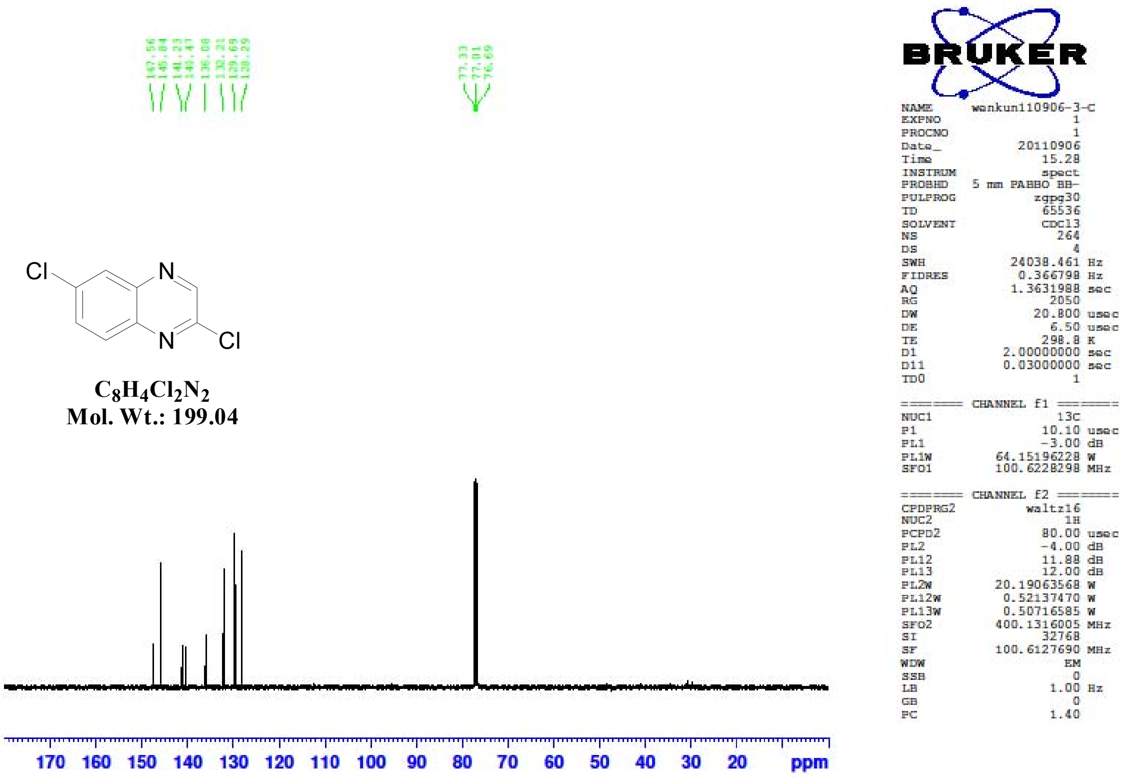

Compound 18b

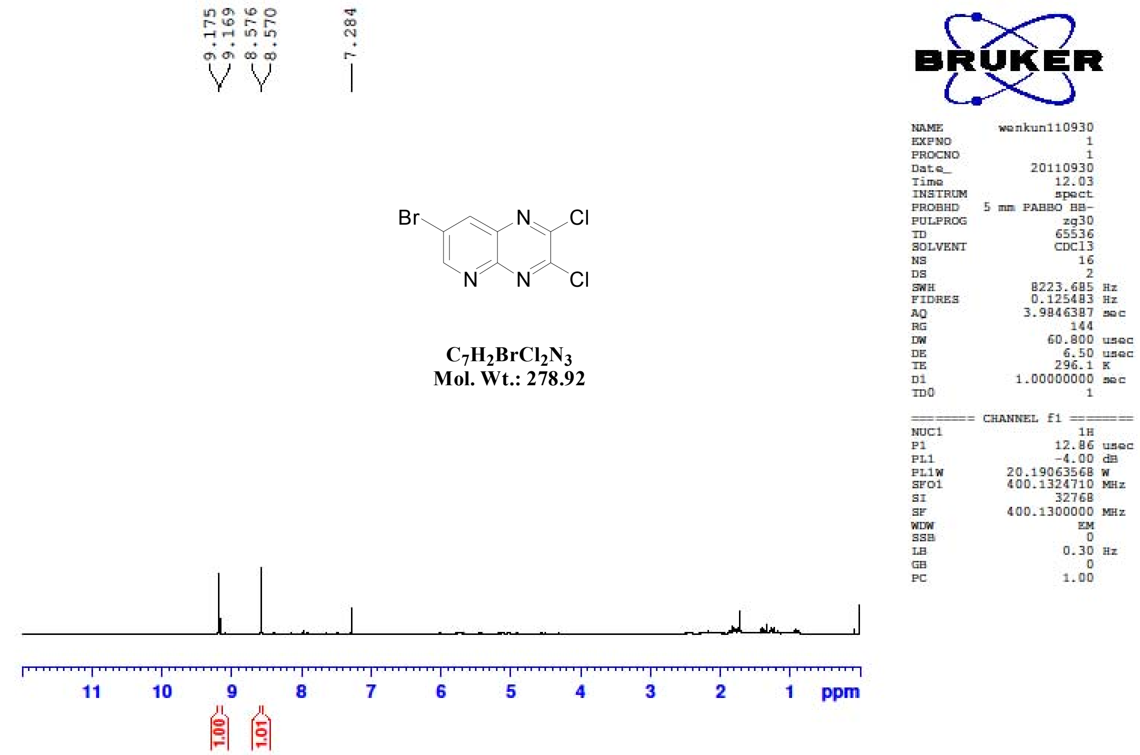

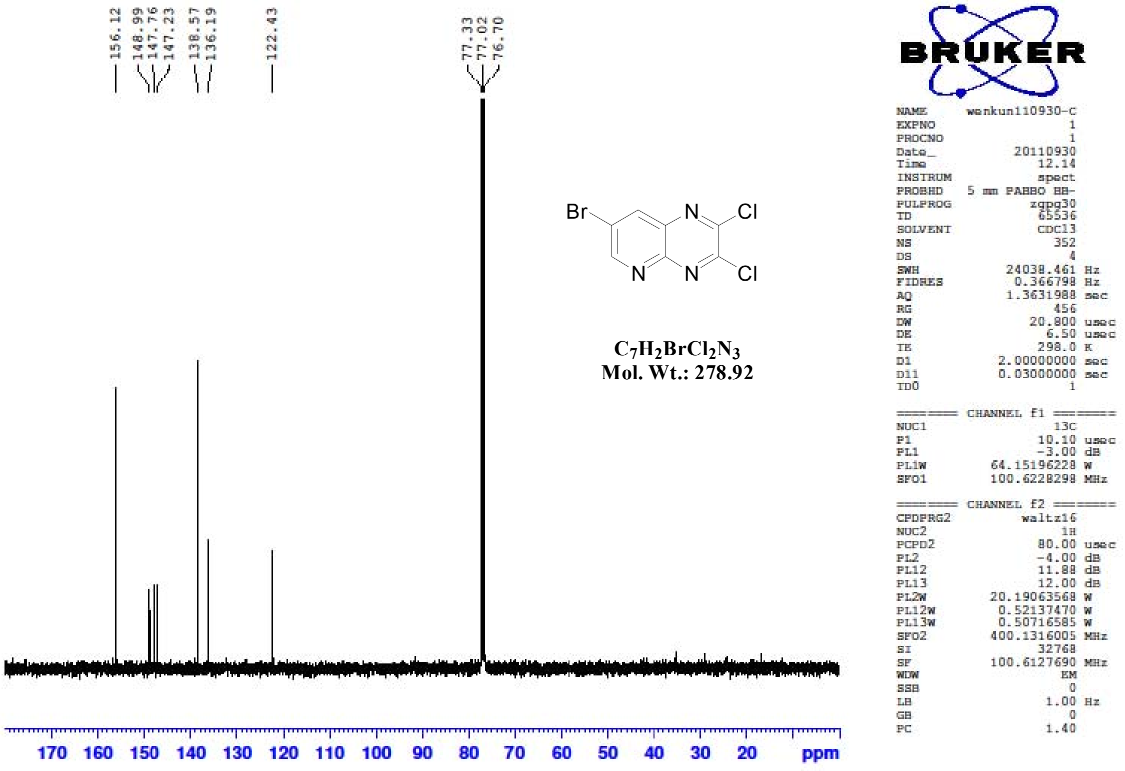

Compound 19: *N*-phenyl-trifluoroacetimidoyl chloride

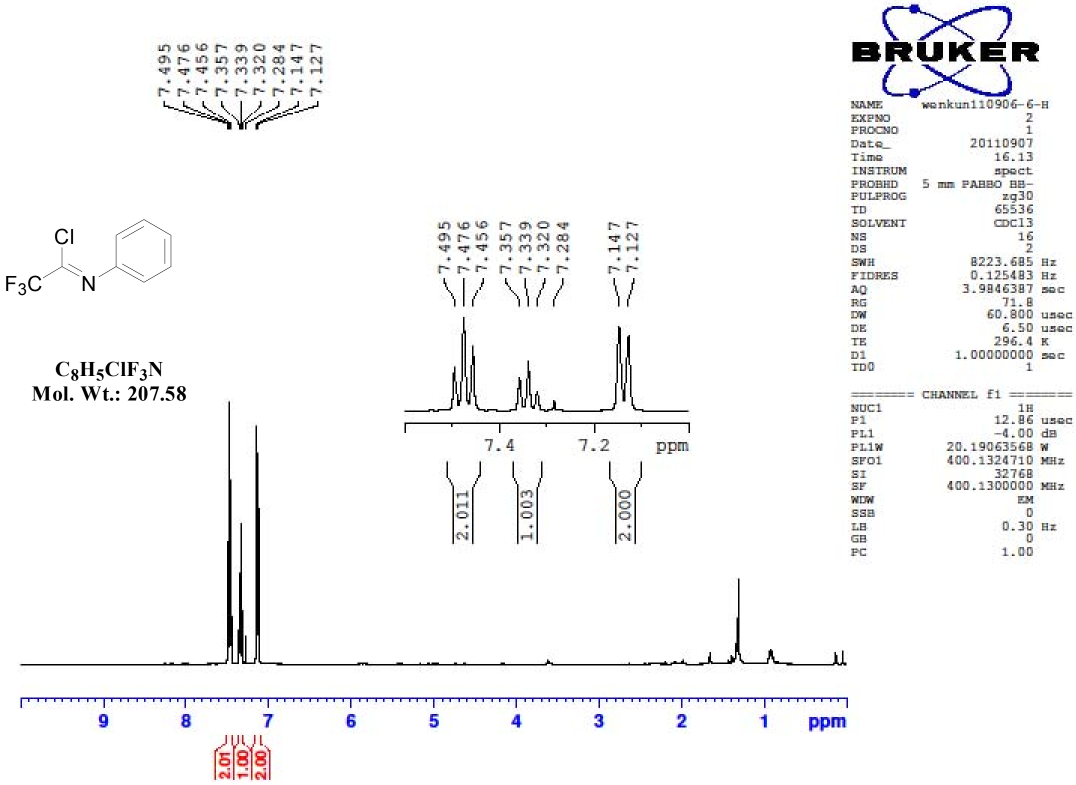

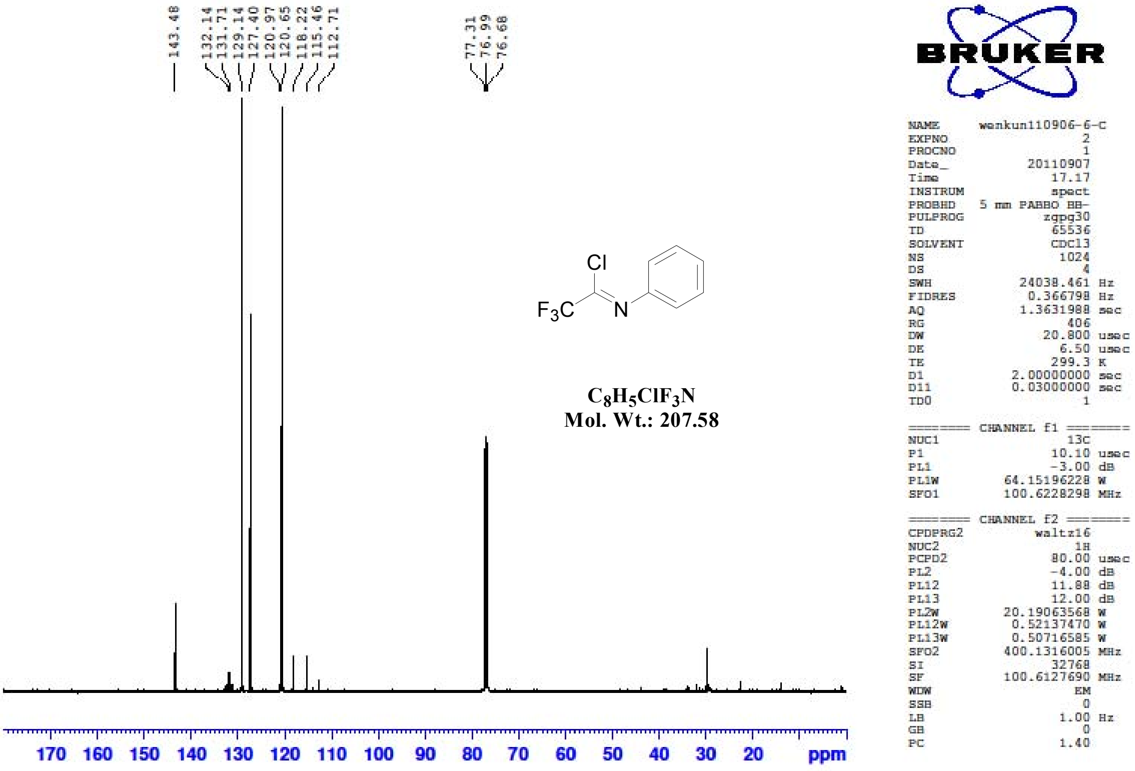

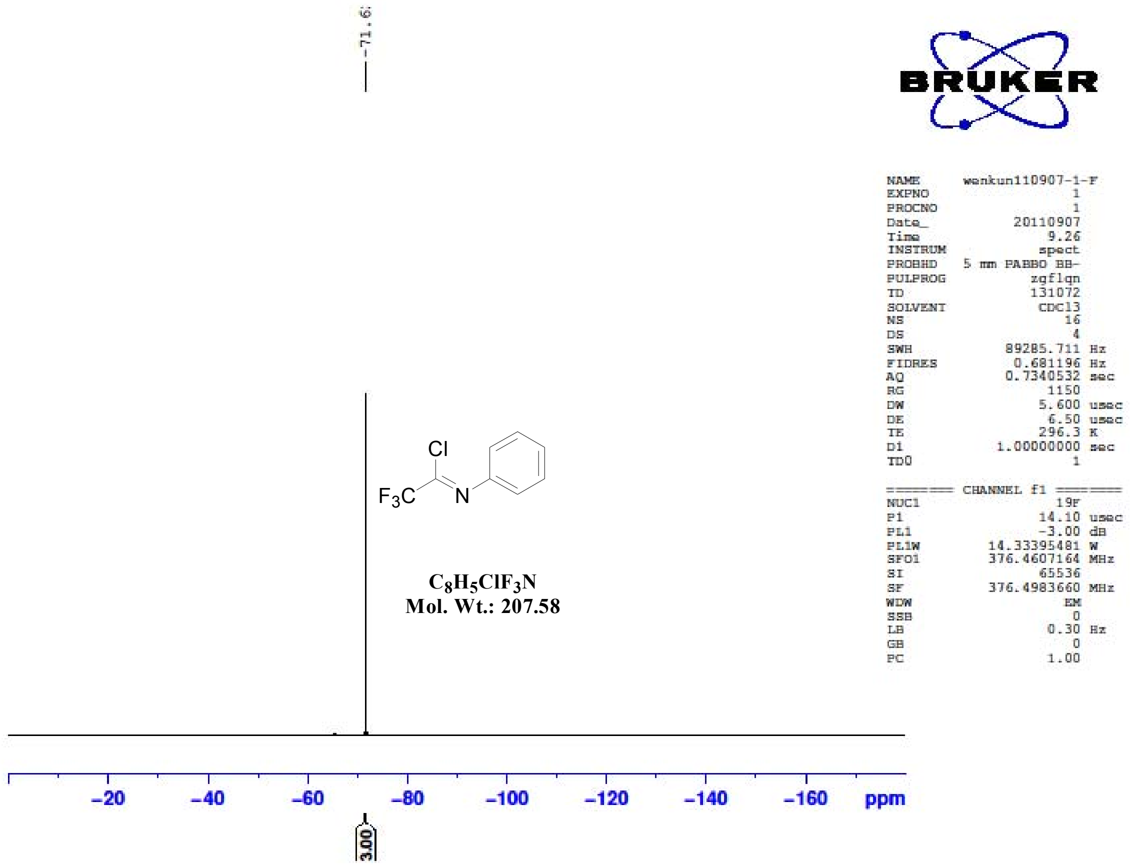

Compound 20: **2,5-Dichloro-3,6-dimethyl-pyrazine**

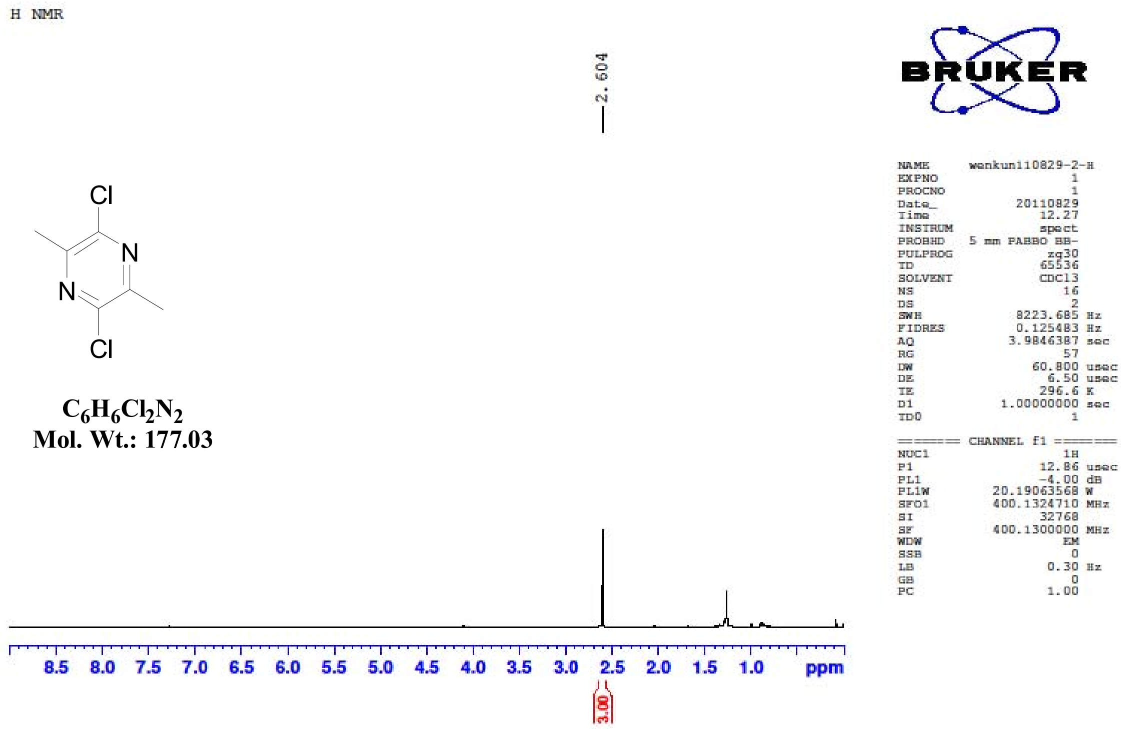

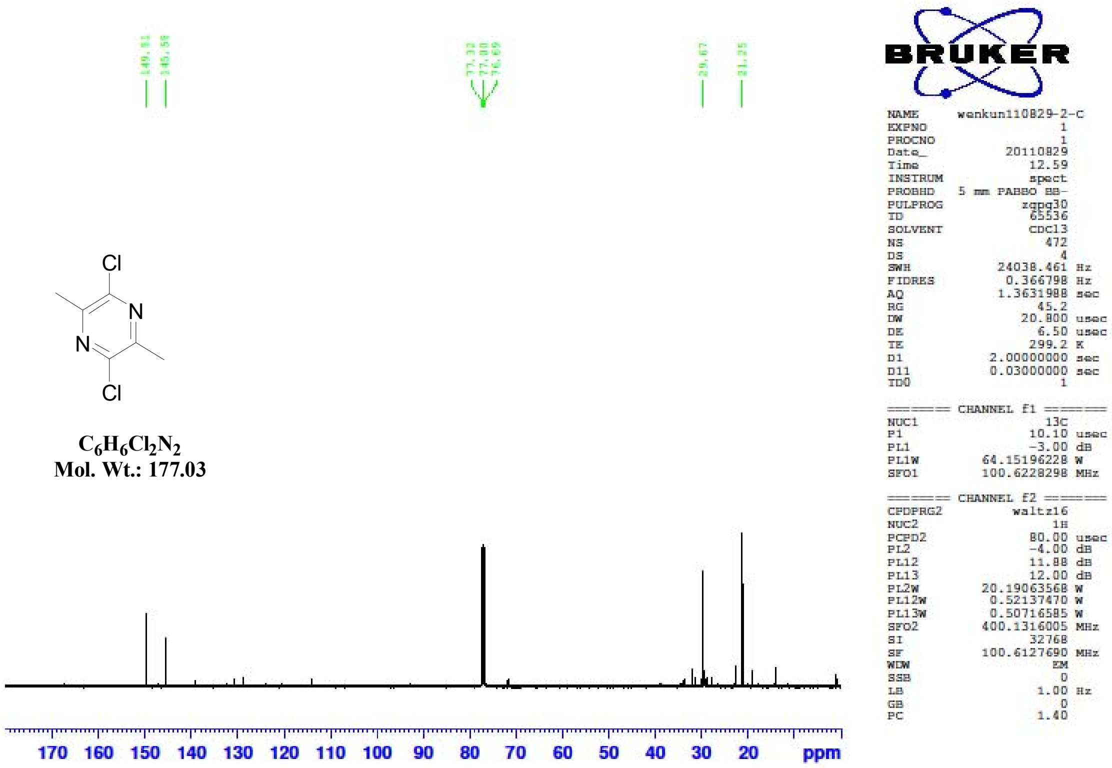

Compound 21: 2,5-Dichloro-3,6-dibenzyl-pyrazine

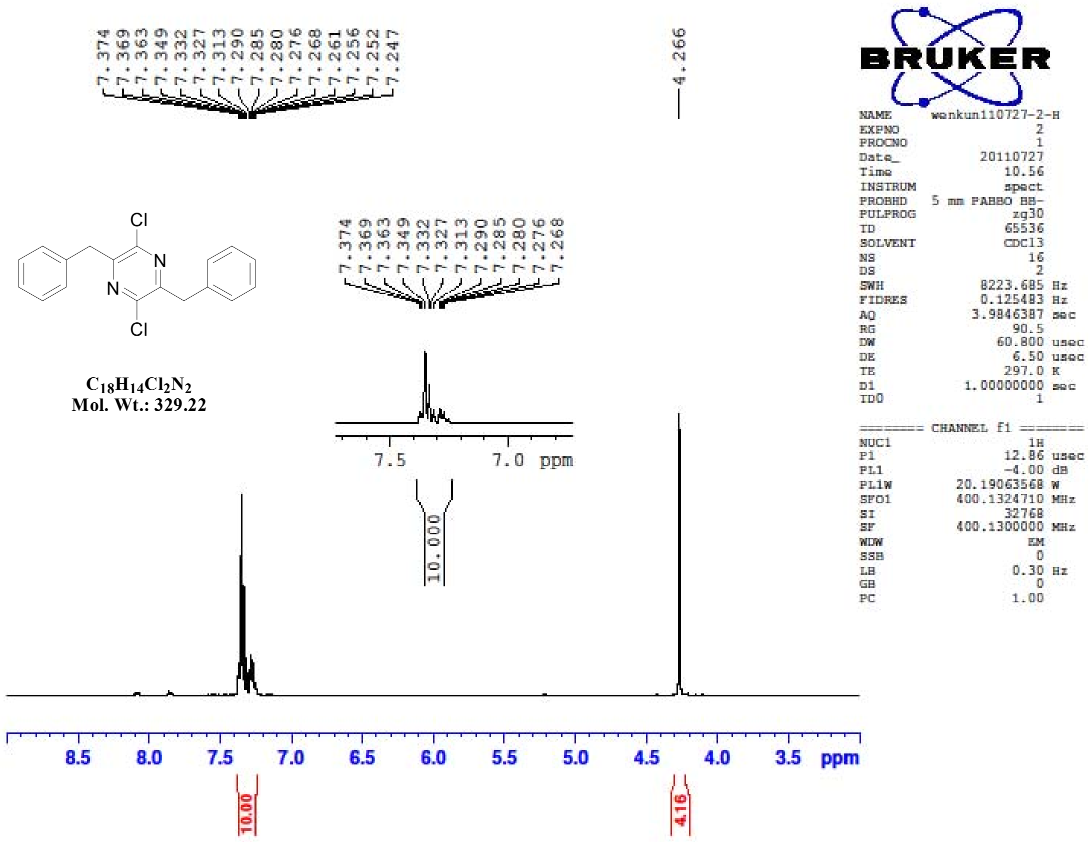

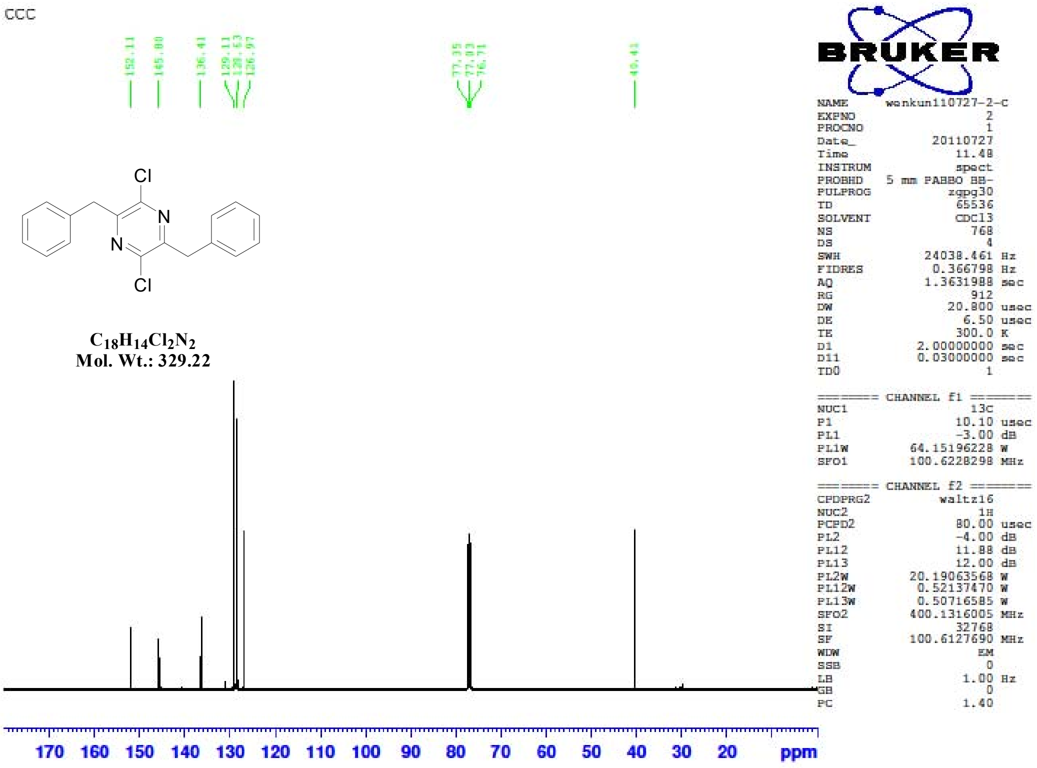
CCC
152.11
145.80
136.41
129.11
128.53
126.97
77.35
77.03
76.71
40.41
Cl
N
N
Cl
C18H14Cl2N2
Mol. Wt.: 329.22
BRUKER
NAME wankun110727-2-c
EXPNO 2
PROCNO 1
Date_ 20110727
Time 11.48
INSTRUM spect
PROBHD 5 mm PABBO BB-
PULPROG zgpg30
TD 65536
SOLVENT CDCl3
NS 768
DS 4
SWH 24038.461 Hz
FIDRES 0.366798 Hz
AQ 1.3631988 sec
RG 912
DW 20.800 usec
DE 6.50 usec
TE 300.0 K
D1 2.00000000 sec
D11 0.03000000 sec
TD0 1
======== CHANNEL f1 ========
NUC1 13C
P1 10.10 usec
PL1 -3.00 dB
PL1W 64.15196228 W
SFO1 100.6228298 MHz
======== CHANNEL f2 ========
CPDPRG2 waltz16
NUC2 1H
PCPD2 80.00 usec
PL2 -4.00 dB
PL12 11.88 dB
PL13 12.00 dB
PL2W 20.19063568 W
PL12W 0.52137470 W
PL13W 0.50716585 W
SFO2 400.1316005 MHz
SI 32768
SF 100.6127690 MHz
WDW EM
SSB 0
LB 1.00 Hz
GB 0
PC 1.40
170 160 150 140 130 120 110 100 90 80 70 60 50 40 30 20 ppm
